# Synthesis and Characterization of a Novel Biphenol-Based Gadolinium Complex for Encapsulation in Human Red Blood Cells

**DOI:** 10.3390/ijms27083492

**Published:** 2026-04-14

**Authors:** Antonella Antonelli, Riccardo Di Corato, Luca Mancini, Michela Cangiotti, Laura Valentini, Luca Giorgi, Gianluca Ambrosi, Pietro Gobbi, Erika Palazzetti, Luigia Rossi, Mauro Magnani

**Affiliations:** 1 Dipartimento di Scienze Biomolecolari (DISB), Università degli Studi di Urbino Carlo Bo, Campus Scientifico “E. Mattei”, Via Cà Le Suore 2-4, 61029 Urbino, Italy; luca.mancini@uniurb.it (L.M.); laura.valentini@uniurb.it (L.V.); pietro.gobbi@uniurb.it (P.G.); luigia.rossi@uniurb.it (L.R.); mauro.magnani@uniurb.it (M.M.); 2 Institute for Microelectronics and Microsystems (IMM), CNR, Via Monteroni, 73100 Lecce, Italy; riccardo.dicorato@cnr.it; 3 Center for Biomolecular Nanotechnologies, Istituto Italiano di Tecnologia, Arnesano, 73100 Lecce, Italy; 4 Dipartimento di Scienze Pure e Applicate (DiSPeA), Università degli Studi di Urbino Carlo Bo, Campus Scientifico “E. Mattei”, Via Cà Le Suore 2-4, 61029 Urbino, Italy; michela.cangiotti@uniurb.it (M.C.); luca.giorgi@uniurb.it (L.G.); gianluca.ambrosi@uniurb.it (G.A.); erika.palazzetti@uniurb.it (E.P.)

**Keywords:** Gd complex synthesis, methodologies, biocompatibility, red blood cells (RBCs), Gd-loaded RBCs

## Abstract

Gadolinium-based contrast agents are widely used in clinical magnetic resonance imaging (MRI) due to their strong paramagnetic properties and ability to enhance image contrast. Despite their diagnostic value, concerns remain regarding gadolinium toxicity and long-term tissue retention, particularly for less stable linear chelates. In this study, we report preliminary results on a newly synthesized gadolinium-based compound (L-Gd), in which the Gd^3+^ ion is coordinated to a specific ligand designed to improve biocompatibility. To evaluate the feasibility of L-Gd encapsulation within human RBCs (hRBCs) for drug delivery, its biocompatibility and cellular interactions were thoroughly investigated. RBCs represent an attractive biomimetic carrier system capable of limiting the direct exposure of tissues to paramagnetic agents while potentially improving circulation time and safety. In vitro assays demonstrated that L-Gd maintains high compatibility with hRBCs within specific concentration ranges, showing no significant hemolysis or morphological alterations. Furthermore, preliminary encapsulation studies indicate that L-Gd can be successfully associated with RBCs, supporting the potential of this approach for contrast agent delivery. These findings suggest that RBC-mediated transport of gadolinium complexes may represent a promising strategy to reduce toxicity and mitigate gadolinium retention. Further investigations will focus on optimizing encapsulation efficiency, relaxometric properties, and in vivo behavior of the L-Gd system.

## 1. Introduction

Gadolinium (Gd)-based contrast agents (GBCAs) are indispensable in clinical magnetic resonance imaging (MRI) due to their potent paramagnetic properties, which significantly enhance image contrast and diagnostic sensitivity. The efficacy of these agents is governed by structural, electronic, and dynamic factors that dictate their relaxivity [1,2]. Upon administration, GBCAs induce marked hyperintensity in target tissues, a phenomenon arising from the seven unpaired electrons of the Gd^3+^ ion. As a lanthanide metal, Gd^3+^ effectively shortens the longitudinal relaxation time (T1) of adjacent water protons, thereby increasing the signal intensity on T1-weighted images [3,4,5]. These interactions improve MRI reliability across various clinical applications, including the imaging of vascular structures, tumors, and regions with compromised blood–brain barrier integrity. Specifically, tumor-specific enhancement is driven by the extravasation of contrast agents through the permeable walls of neo-vessels associated with tumor angiogenesis [6,7].

Given the inherent toxicity of free Gd^3+^ ions, which can disrupt calcium-dependent biological pathways, GBCAs are formulated as chelated complexes where the metal ion is sequestered by organic ligands [8]. These agents are classified by their ligand architecture and ionic character into four main categories: linear-ionic, linear-nonionic, macrocyclic-ionic, and macrocyclic-nonionic (Table 1). The chemical structure of the ligand determines the thermodynamic and kinetic stability of the Gd(III) complex, directly influencing its in vivo safety and renal clearance profile [9,10,11,12,13]. Linear agents, such as gadopentetate dimeglumine, gadobenate dimeglumine, and gadodiamide, typically exhibit lower kinetic stability and a higher risk of gadolinium dissociation, particularly in patients with impaired renal function [14]. In contrast, macrocyclic agents encapsulate the Gd^3+^ ion within a rigid, ring-like structure, significantly reducing ion release and the risk of adverse effects such as nephrogenic systemic fibrosis (NSF) [15,16,17,18].

Despite their clinical utility, GBCAs are under renewed scrutiny regarding gadolinium retention and emerging toxicity profiles. In renally compromised patients, Gd exposure is linked to NSF, a severe fibrosing disorder [19,20]. Furthermore, recent evidence indicates that trace amounts of gadolinium may persist in the brain, bone, and other tissues, even in individuals with normal renal function [21,22]. While a definitive causal relationship between retention and adverse neurological outcomes remains unproven, these observations have prompted increased regulatory oversight. Consequently, the European Medicines Agency (EMA) has restricted the use of several linear GBCAs due to their propensity for gadolinium release and tissue deposition [23]. While novel high-relaxivity macrocyclic agents like gadopiclenol offer improved safety at lower doses (0.05 mmol/kg) [24,25,26], the persistence of gadolinium in the body highlights the urgent need for safer formulations to mitigate long-term risks associated with complex retention mechanisms [27,28,29,30].

**Table 1:** FDA approved Gd-based contrast agents.

Brand Name	Chemical Name	Ligand Type	Structure Type	Ionic Character	Notes	Refs.
Dotarem/Clariscan	Gadoterate meglumine (Gd-DOTA)	DOTA	Macrocyclic	Ionic	High kinetic stability	[1,2,8]
Eovist/Primovist	Gadoxetate disodium (Gd-EOB-DTPA)	EOB-DTPA	Linear	Ionic	Liver-specific uptake	[1,12,16,19]
Gadavist	Gadobutrol (Gd-BT-DO3A)	BT-DO3A	Macrocyclic	Non-ionic	High relaxivity	[1,8,9,19]
MultiHance	Gadobenate dimeglumine (Gd-BOPTA)	BOPTA	Linear	Ionic	Partial hepatobiliary excretion	[1,5,12,19]
Omniscan	Gadodiamide (Gd-DTPA-BMA)	DTPA-BMA	Linear	Non-ionic	Restricted use (EMA)	[6,15,18,19,22]
OptiMARK	Gadoversetamide (Gd-DTPA-BMEA)	DTPA-BMEA	Linear	Non-ionic	Suspended (EMA)	[6,15,18,19,22]
ProHance	Gadoteridol (Gd-HP-DO3A)	HP-DO3A	Macrocyclic	Non-ionic	High kinetic; thermodynamic stability	[1,11,19]
Elucirem/Vueway	Gadopiclenol	-	Macrocyclic	Non-ionic	Recently approved; enhanced relaxivity	[13,23,24,25,26]

In this context, the present study reports preliminary findings on a newly synthesized gadolinium-based compound, GdH_3_L (L-Gd), featuring a specifically designed coordination ligand. The biocompatibility of L-Gd was evaluated using human red blood cells (hRBCs) as a biomimetic cellular model, alongside initial in vitro investigations of its encapsulation within RBCs. The utilization of RBCs as a biomaterial-based delivery system represents a promising strategy for the transport of Gd-containing agents [31,32]. By acting as a biocompatible carrier, RBC encapsulation may shield the paramagnetic compound from biological tissues, thereby enhancing its safety profile and suitability for advanced diagnostic imaging applications.

## 2. Results

### 2.1. Synthesis of the Gadolinium Complex

**Figure 1:**
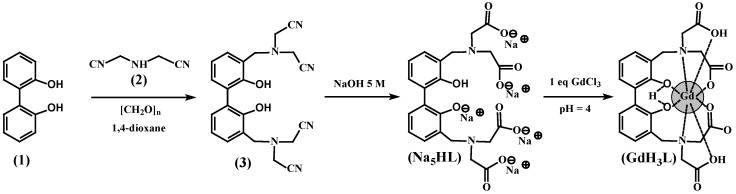
Synthetic scheme for the [GdH_3_L] complex (L-Gd).

The ligand (H_6_L) and its corresponding gadolinium complex (GdH_3_L, designated as L-Gd) were synthesized according to previously reported methods (Figure 1). The synthesis involves a Mannich condensation reaction of bisphenol, paraformaldehyde, and iminodiacetonitrile using 1,4-dioxane as the solvent to give the intermediate (3) [33]. Subsequent hydrolysis leads to the formation of the ligand (Na_5_HL) as sodium salt. The structures of the intermediate and the ligand were confirmed by ^1^H NMR, ^13^C NMR, and FTIR spectra. The spectra of all compounds synthesized and shown in Figure 1 are provided in the Supplementary Materials (Figure S1).

This ligand framework is expected to stabilize Gd^3+^ ions in pseudo-dodecahedral or bicapped trigonal prismatic geometries. These coordination motifs are known to confer kinetic inertness, thermodynamic stability, and restricted inner-sphere water exchange, all of which are advantageous for MRI contrast enhancement [33].

### 2.2. Characterization of L-Gd Compound

To confirm the successful coordination of the Gd^3+^ ion and to evaluate the structural and chemical properties of the resulting complex, a multi-technique characterization approach was employed. This included vibrational spectroscopy for coordination geometry, elemental analysis for composition, and magnetic resonance techniques to probe the electronic environment.

#### 2.2.1. IR Spectroscopic Study

**Figure 2:**
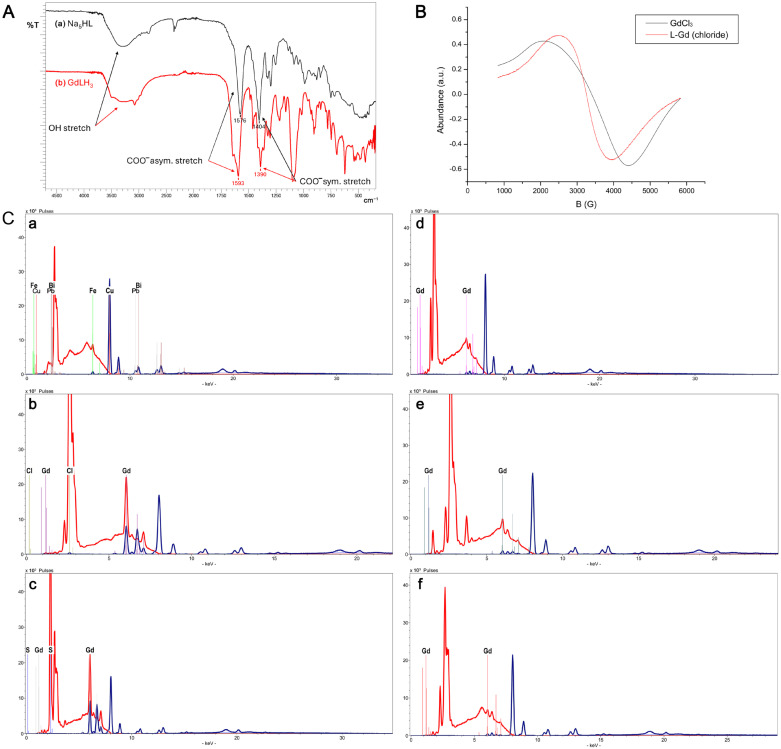
(**A**) IR spectra of the ligand Na_5_HL (**a**) and GdH_3_L complex (**b**). (**B**) Normalized X-band EPR spectra of GdCl_3_ and the L-Gd complex recorded in the solid state at 298 K. Spectra were normalized to the same peak intensity to allow for a direct comparison of line shape and linewidth. The magnetic field is reported on the x-axis (G), and the first-derivative EPR signal intensity (a.u.) on the y-axis. (**C**) ED-XRF analyses: (**a**) Spectrum performed on the tape glued to the metal stub without any samples (8 kV in red, 40 kV in green), (**b**) sample spectrum GdCl_3_ (red 8 kV, green 40 kV), (**c**) sample spectrum of Gd_2_(SO_4_)_3_ (red 8 kV, green 40 kV), (**d**) spectrum of the 1st batch of L-Gd complex (red 8 kV, green 40 kV), (**e**) spectrum of sterilized 1st batch of L-Gd complex (in red 8 kV, in green 40 kV), and (**f**) spectrum of 2nd batch of L-Gd complex (red 8 kV, green 40 kV).

Initially, FTIR spectroscopy was performed to provide direct evidence of ligand coordination by monitoring shifts in characteristic functional group vibrations [34]. As shown in Figure 2, a comparison between the infrared spectra of the Na_5_HL ligand (Figure 2A(a)) and the GdH_3_L complex (Figure 2A(b)) revealed significant shifts in the characteristic carboxylate absorption bands, confirming the formation of the metal complex. The spectra of the Na_5_HL ligand were characterized by the peak at 1576 cm^−1^ due to the asymmetric stretching vibration of COO and at 1404 cm^−1^ due to the symmetric stretching vibration of COO. After complexing with Gd^3+^, the positions of the peak at 1576 cm^−1^ shifted to 1593 cm^−1^ while the position of the peak at 1404 cm^−1^ shifted to 1390 cm^−1^, indicating that coordination took place. Moreover, it is known that the carboxylate group has three types of coordination (unidentate, bidentate or bridge). Based on this criterion, unidentate coordination is indicated when the difference in frequencies between asymmetric and symmetric stretching vibrations [∆ν = (ν_asym_ COO– − ν_sym_ COO–)] ≥ 200 [35]. From the infrared spectra of the Gd(III) complex, the calculated ∆ν was 203 cm^–1^, which suggests a unidentate interaction of the carboxylate group toward the Gd ions in the complex.

Independent syntheses of the GdH_3_L complex were performed to validate the preparation protocol, followed by FTIR spectroscopic analysis. The resulting spectra from different batches (e.g., 1st and 2nd batch) were identical, thereby confirming the reproducibility of the synthetic route. Elemental analysis was performed on both batches, confirming that their purity was compatible. Anal. Calc. for C_22_H_23_GdN_2_O_11_ (648.7): C, 40.7%; H, 3.6%; N, 4.3%. Found for batch 1: C, 40.8%; H, 3.7%; N, 4.2%. Found for batch 2: C, 40.9%; H, 3.5%; N, 4.2%. The L-Gd powder was sterilized at 120 °C for 24 h to assess its functionality in a cell-based drug delivery model, specifically using human red blood cells (hRBCs) under sterile conditions. FTIR spectroscopy was performed on two samples: one before and one after sterilization. The overlaid spectra were identical, indicating that the complex remained intact and confirming its thermal stability, Figure S2.

#### 2.2.2. ED-XRF Analyses

While FTIR confirmed the involvement of carboxylate groups in metal binding, ED-XRF analysis was subsequently employed to verify the elemental composition and ensure the presence of gadolinium within the synthesized powder across different batches. Gadolinium peaks obtained from the ED-XRF spectra of GdCl_3_, GdSO_4_ powders as well as of the 1st and 2nd batches of L-Gd complex derived from GdCl_3_ salt, were observed in all analyses and confirmed by the reference spectrum of 99% pure gadolinium (Figure S3). These analyses are shown in Figure 2C(b–f) and compared to the control spectrum (Figure 2C(a)) acquired from the adhesive tape attached to the metal stub without any sample. Notably, sterilization of the L-Gd complex powder at 120 °C for 24 h did not result in any spectra changes, as expected.

#### 2.2.3. EPR Measurements

To complement the structural information obtained from FTIR and elemental data, Electron Paramagnetic Resonance (EPR) spectroscopy was utilized to probe the electronic environment and coordination features of the paramagnetic Gd^3+^ center. This technique provides specific insight into the magnetic microenvironment, which is crucial for potential MRI applications. In DMSO solution, the L-Gd complex exhibited a single broad isotropic resonance centered at g ≈ 2.05, without resolved hyperfine structure, consistent with the typical behavior of Gd^3+^ ions in the ^8^S_7_/_2_ ground state. The absence of hyperfine features reflects fast electronic relaxation and efficient motional averaging in solution. The experimental g value, slightly shifted from the free-electron value (g = 2.0023), is consistent with the presence of a ligand field surrounding the Gd^3+^ center, as commonly observed for clinically relevant Gd(III) chelates [24,33,36,37,38]. The multidentate ligand L, containing both oxygen and nitrogen donor atoms, supports the formation of highly coordinated Gd^3+^ species. Although EPR alone does not allow for unambiguous assignment of the coordination geometry, the observed spectral features are compatible with high-coordination environments typically reported for stable Gd(III) complexes used in MRI applications. In the solid state, the EPR spectrum of L-Gd shifted to g ≈ 2.07 and displayed markedly broader and slightly distorted lines. This behavior is characteristic of increased magnetic anisotropy and static disorder in the absence of molecular tumbling. The spectral profile suggests a distribution of ligand-field environments around the Gd^3+^ center. Importantly, the absence of well-resolved dipolar features supports the predominance of magnetically isolated Gd^3+^ centers rather than extended aggregates. For comparison of line shapes, spectra were normalized after parameter determination (Table 2).

In the solid state, the L-Gd complex exhibited significantly narrower linewidths than the reference salts. In particular, ΔB values for L-Gd were lower than those observed for GdCl_3_, indicating reduced dipolar broadening. This behavior is consistent with efficient coordination of Gd^3+^ by the organic ligand, which increases spatial separation between paramagnetic centers and reduces interionic interactions. The g_centroid parameter provides a more representative description of the overall spectral position, especially for asymmetric signals. For L-Gd in DMSO, the difference between g_medium (2.0488) and g_centroid (2.0888) indicates increased crystal-field heterogeneity relative to the reference salt. In contrast, free GdCl_3_ in DMSO showed more symmetric spectra with nearly constant linewidths (384–388 G), consistent with a more homogeneous electronic environment. EPR spectra of GdCl_3_ and the L-Gd complex in solid state (Figure 2B) were acquired under identical instrumental conditions to enable direct comparison. Because EPR signal intensity is proportional to the concentration of EPR-active Gd^3+^ species, spectra of GdCl_3_ solutions at known concentrations were used to construct a calibration curve (Figure S4). The calibration showed an overall linear dependence of integrated intensity on concentration over the 0.2–1.0 mM range. Interpolation of the L-Gd integrated intensity through the calibration equation yielded an estimated amount of paramagnetic Gd corresponding to 2.25 × 10^−3^ mol and an apparent molecular weight of 622.22 g/mol. The calculated apparent concentrations (4.84–7.50 mM) were slightly higher than the nominal value (4.67 mM), which may reflect minor instrumental or matrix effects. Nevertheless, the data indicate an efficient transfer of Gd^3+^ into the homogeneous phase and confirm the presence of EPR-active, well-coordinated species in DMSO. Line-shape analysis further highlights differences between the salt and the complex. GdCl_3_ in DMSO exhibited relatively sharp and symmetric signals, whereas L-Gd showed broader lines (ΔB 779 G) and a larger separation between g_medium and g_centroid, consistent with increased crystal-field perturbation induced by ligand coordination. These linewidth differences reflect the combined effects of solvation and static disorder in the solid state. Additional insight was obtained by comparing complexes prepared from different precursors (GdCl_3_ and Gd_2_(SO_4_)_3_. Relative to the free salts, L-Gd complexes derived from both precursors displayed narrower linewidths (1440–1600 G) and g values in the 2.035–2.076 range, indicating effective metal binding and a more defined coordination environment. Notably, the chloride-derived complex exhibited sharper and more symmetric solid-state spectra, whereas the sulfate-derived sample showed broader and more distorted features (Figure S5). This behavior suggests that chloride is more readily displaced during complex formation, allowing more complete ligand coordination, while sulfate may partially compete for metal binding and introduce greater structural heterogeneity. Remarkably, ICP-OES analysis was found to be in excellent agreement with theoretical EPR data regarding the molecular weight (MW) of the newly synthesized L-Gd complex (assuming a 1:1 Gd-to-ligand stoichiometry). Specifically, the MW calculated from the Gd content in the L-Gd powder (0.243 mg of Gd per mg of powder) was determined to be 642.2 Da, consistent with the predicted molecular structure.

#### 2.2.4. ESEM-EDS Analysis of GdCl_3_ and the L-Gd Complex

Finally, the morphological stability and chemical homogeneity of the complex were assessed at the microscale using ESEM-EDS. This analysis was particularly important to confirm that the sterilization process required for subsequent cell-based studies did not alter the chemical integrity of the compound. Representative ESEM-EDS images of reproducible L-Gd powders (synthesized from GdCl_3_) and their corresponding spectra are shown in Figure S6. This complex was selected for all subsequent experiments reported herein due to its superior EPR characteristics. For comparison, ESEM analyses of the GdCl_3_ precursor alone (Figure S7) are also provided in the Supplementary Materials.

### 2.3. Testing Cell Biocompatibility of L-Gd Compound

#### 2.3.1. In Vitro Biocompatibility of L-Gd in HEK293 Cells

To assess the potential cytotoxicity of the Gd–ligand complex, human renal cells, specifically Human Embryonic Kidney (HEK) 293 cells were used because they represent a well-characterized human cell line commonly used for basal toxicity screening. Using a non-cancerous immortalized model allows for an accurate evaluation of how the compound interacts with standard cellular processes, ensuring a more relevant assessment of its safety profile in a human context. Results were compared with control cells, including untreated HEK 293 cells and HEK 293 cells treated with 50 µL of DMSO alone. The viability percentage (66 ± 0.60%), average cell diameter (13.41 µm), and average viable cell diameter (15.82 µm) of L-Gd-treated cells were comparable to those of the control cells (untreated HEK 293 cells: 69.6 ± 0.78% viability, average diameter 13.3 µm, average viable diameter 14.75 µm; DMSO-treated HEK 293 cells: 66.8 ± 0.84% viability, average diameter 13.4 µm, average viable diameter 15.81 µm). No significant differences in cell viability were observed between the DMSO and L-Gd treatments (*p*-value = 0.28). However, both the DMSO and L-Gd groups showed significant differences compared to the control cells (respectively, *p*-values of 0.0139 and 0.0035), which can be attributed exclusively to the effect of the solvent.

#### 2.3.2. Preliminary Biocompatibility Assessment of L-Gd Complexes with Human RBCs (hRBCs)

Prior to L-Gd complex encapsulation, we first evaluated the impact of gadolinium on human erythrocyte integrity by incubating the cells with either free GdCl_3_ or the L-Gd complex at room temperature for 3 h under gentle agitation as described in the Supplementary Materials. Cellular biological parameters were evaluated using a hemocytometer at t = 0 and t = 3 h after incubation. As shown in Table S1, the native RBC parameters did not exhibit significant changes after 3 h of incubation with GdCl_3_. Table S1 also reports the NMR longitudinal T1 and transverse T2 relaxation times measured at the end of the 3-h incubation period, before and after washing of the cell suspension to evaluate the overall paramagnetic effect in the samples. Transmission electron microscopy (TEM) analyses were performed on the same samples. Representative images of human RBCs incubated with GdCl_3_, either washed (A) or unwashed (B) with HEPES buffer and untreated or control RBCs (C) are shown in Figure S8. The analyses indicate that both samples preserve the typical morphological features of red blood cells, suggesting that the metal salt does not induce detectable cellular alterations.

Following the initial assessment reported below, the biocompatibility of the newly synthesized L-Gd complex with hRBCs was evaluated under similar conditions. The results are summarized in Table S2 alongside the relaxation times measured by 400 MHz NMR, which were used only to validate the presence of gadolinium in decreasing relaxation times relative to control samples. Moreover, data obtained by testing the addition of the L-Gd complex to whole blood (w.b.) at a physiological hematocrit (approximately 44%) were also reported. Notably, no changes in the typical biological properties of RBCs were observed (Table S2). Furthermore, transmission electron microscopy (TEM) analysis (Figure S9) revealed normal RBC morphology after incubation with L-Gd (Figure S9A), with no detectable intra- or extracellular alterations compared to untreated control RBCs (Figure S9B).

### 2.4. Experimental Loading of L-Gd Complex in the hRBCs

Preliminary experiments were performed to evaluate the encapsulation of the L-Gd complex within human RBCs. To evaluate the reproducibility of the chemical synthesis, 1 mg of two independent batches of L-Gd (1st and 2nd batch), each dissolved in 50 µL of DMSO were loaded into human RBCs under identical conditions (1 mg L-Gd/500 µL RBCs at 70% Hct). The loading procedure was carried out according to the protocol described in Section 4. The biological parameters of the resulting samples, resuspended in HEPES buffer at a physiological hematocrit (44%), were assessed using a hemocytometer. The values were compared to those of the control cells, which were unloaded RBCs (UL-RBCs) prepared following the same procedure with the exception that they were dialyzed in the absence of L-Gd complex (Table 3). Furthermore, NMR measurements showed a significant decrease in T_1_ and T_2_ relaxation times (peak intensity) with respect to the control values, confirming the presence of the L-Gd complex in both final samples.

**Figure 3:**
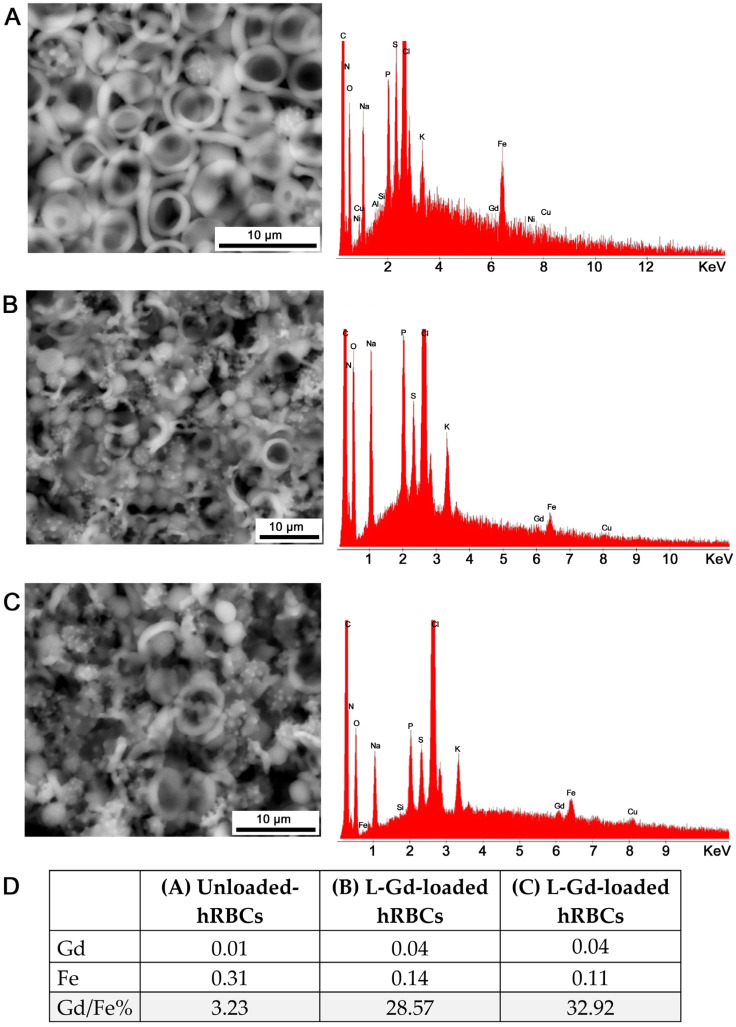
ESEM-EDS images and corresponding spectra of (**A**) control unloaded hRBCs, and L-Gd loaded hRBC samples prepared with the (**B**) first and (**C**) second batch of the complex. The images were acquired at slightly different magnifications. The EDS analyses were performed under the same analytical conditions. (**D**) Gd/Fe percentage ratio calculated from at least ten ESEM-EDS acquisitions per sample (A, B, and C samples).

ESEM-EDS analysis (Figure 3) of L-Gd-loaded RBCs compared with unloaded control RBCs confirmed the intracellular presence of gadolinium. Since the aim of the study was to detect the presence of Gd within the cells, RBCs were analyzed twice by focusing the electron beam on a small area of the cell. The first analysis was performed at the maximum accelerating voltage of the incident beam (30 kV) with a counting time of 100 s. The second analysis was carried out at the same accelerating voltage and on the same area, but with a prolonged counting time of up to 500 s. Notably, under these analytical conditions, the electron beam induces localized degradation or ‘perforations’ in the cell structure. This degradation allows for the verification of Gd both on the cell surface and at the intracellular level once the outer membrane has been ablated. Figure 3 presents backscattered electron (BSE) images of the analyzed cells alongside their corresponding spectra (displayed to the right of each image). Image (A) shows the control sample (unloaded RBCs), where the spectrum confirms the absence of Gd, while (B) and (C) represent L-Gd-loaded hRBCs, with their respective spectra clearly indicating the presence of Gd. Table 4 presents the average semi-quantitative atomic percentages of Gd and Fe, calculated from at least ten acquisitions per sample, with the Gd/Fe% ratio also reported.

**Figure 4:**
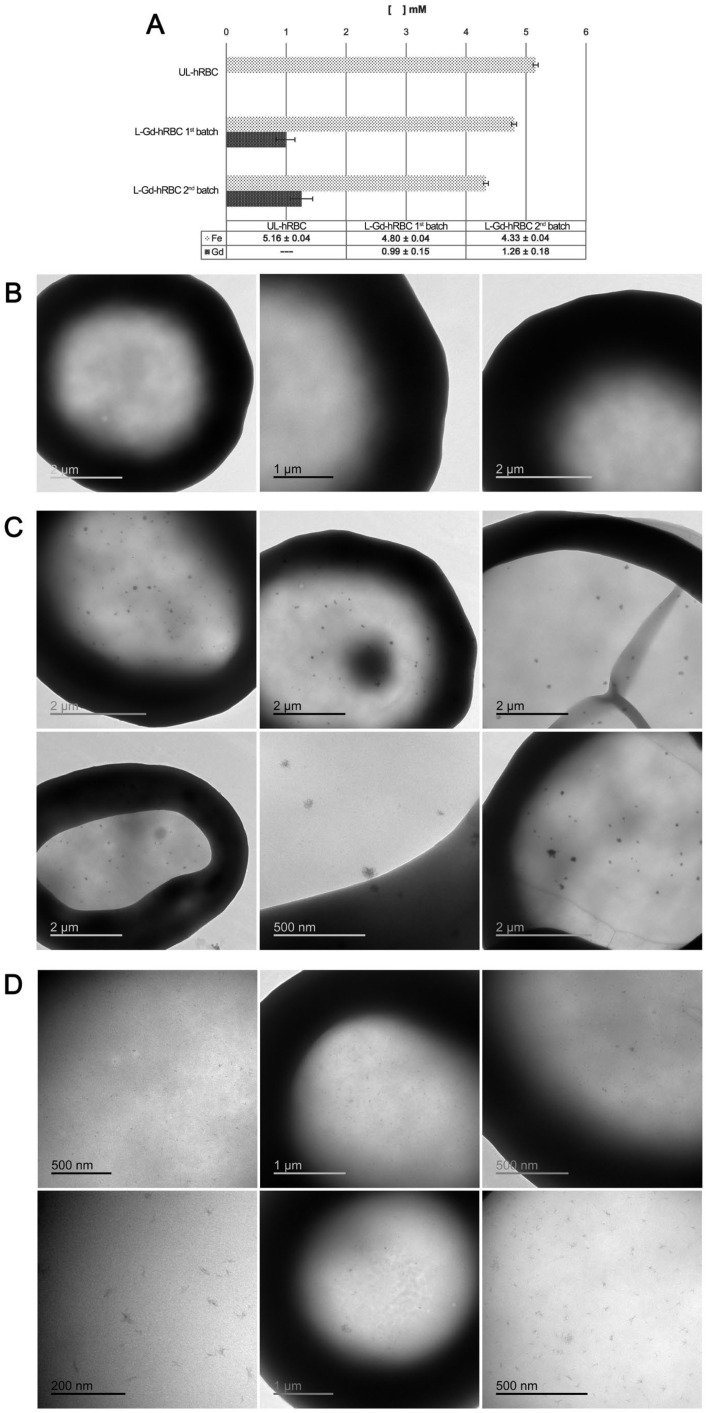
(**A**) ICP analysis of human L-Gd-loaded RBCs versus unloaded control samples (hRBCs-UL) after the loading procedure. Representative TEM images of (**B**) control unloaded RBCs (UL-RBCs) and L-Gd-loaded RBCs prepared using the first (**C**) and second batch (**D**) of the complex.

As shown in Figure 4, the analytical characterization confirms the successful internalization of the complex. Specifically, ICP-OES measurements (Figure 4A) revealed a significant increase in gadolinium (Gd) content, accompanied by a reduction in iron percentage compared to the control unloaded RBCs (UL-RBCs). The data clearly demonstrate that Gd atoms were quantifiable in both loaded samples, with final intracellular concentrations ranging between 1.0 and 1.3 mM, well above the detection limit. The observed decrease in iron content in L-Gd-loaded hRBCs is likely due to a partial loss of hemoglobin during the hypotonic dialysis step, a finding further investigated in the subsequent loading experiments. Furthermore, Figure 4 reports the TEM analysis performed to evaluate cell morphology and internal structure. The images confirm that the native physical and physiological characteristics of the hRBCs remained unaltered, with cells exhibiting their typical biconcave shape and an intact membrane. In contrast to the control cells, L-Gd-loaded hRBCs displayed electron-dense clusters dispersed within the cytoplasm, representing the entrapped gadolinium complex. These findings were further validated by ICP-OES measurements performed on the same experimental samples, and the results are presented in Figure 4A. These observations were consistent for samples prepared using both the first and second L-Gd complex batches, which were loaded under identical experimental conditions. During these evaluations, we also assessed the ligand (L) alone to determine whether its encapsulation within human RBCs induces morphological alterations or affects biological parameters. Specifically, the final set of experiments involved applying the same loading procedure to RBCs treated with either the ligand alone or the L-Gd complex. This finding was further investigated through additional loading experiments, which are described in the following section.

#### 2.4.1. Assessment of Ligand (L) Encapsulation in hRBCs Using the L-Gd Loading Protocol

Initially, a preliminary 24 h incubation of hRBCs with the ligand alone was performed to evaluate its individual effect. RBCs separated from whole blood were washed in HEPES buffer and adjusted to a hematocrit (HCT) of 70%, matching the conditions used during the dialysis step of the loading protocol. No significant differences in typical biological parameters were observed compared to the control RBCs under the same conditions. These results indicate that prolonged incubation with the ligand (L) does not affect erythrocyte indices. During these evaluations, we also assessed the encapsulation of the ligand (L) alone to determine whether its use induces morphological alterations or affects the biological parameters of hRBCs. Specifically, this set of experiments involved applying the same loading procedure to RBCs treated with either the ligand alone (1 mg dose initially used) or the L-Gd complex prepared using 1 and 2 mg doses (L1-Gd-loaded RBCs and L2-Gd-loaded RBCs, respectively). To ensure identical experimental conditions, an equal volume of DMSO (used as the solvent for the L-Gd complex) was added to both the unloaded and ligand-only RBC suspensions during the loading procedure. This confirms that any observed effects are attributable to the active compounds rather than the vehicle itself. At the end of the procedure, all samples were resuspended to a similar hematocrit percentage and evaluated using a hemocytometer to compare the parameters of L-Gd- and ligand-loaded RBC samples with those of the unloaded RBCs (Table 4). No significant differences were found with respect to the control values. Moreover, T1 and T2 relaxation times were determined via ^1^H NMR spectroscopy through peak intensity analysis for unloaded, L1-2-Gd-loaded, and ligand-loaded RBCs (Table 4). As expected, the loading procedure applied to the ligand alone did not lead to any reduction in relaxation times, which remained comparable to those of the control (unloaded RBCs). Conversely, complex encapsulation significantly shortened the T1 and T2 values; this reduction was dose-dependent, with a more pronounced effect observed in L2-Gd-loaded RBCs prepared at twice the concentration relative to L1-Gd-loaded RBCs.

ICP and TEM analyses confirmed that the encapsulation of the ligand alone into hRBCs did not induce significant morphological or intracellular alterations compared to the control (unloaded) RBCs. Specifically, ICP measurements showed that the iron (Fe) content in ligand-loaded hRBCs (3.76 ± 0.09 mM) was comparable to that of unloaded cells (3.32 ± 0.08 mM), as shown in Figure S10. Notably, ICP analysis confirmed the presence of Gd atoms in L-Gd-loaded RBC samples, with the Gd concentration in L2-Gd samples being approximately twofold higher than in the L1-Gd samples (3.43 ± 0.11 mM vs. 1.73 ± 0.08 mM, respectively). Semi-quantitative ESEM-EDS analysis of the atomic and weight percentages for Gd and Fe was conducted on the same sample preparations, with the results summarized in Table 5. Notably, Gd was not detected in either the ligand-loaded RBCs or unloaded RBC (control) samples. Conversely, Gd was consistently identified in RBCs loaded with both L1-Gd and L2-Gd, exhibiting a clear dose-dependent trend in intracellular accumulation.

Moreover, TEM analysis revealed that the loading procedure for the ligand alone did not alter cell morphology, with RBCs exhibiting a size and shape comparable to those of the control (unloaded) cells. In contrast, L-Gd-loaded RBCs displayed prominent intracellular clusters, attributable to the entrapment of the gadolinium complex, consistent with our previous observations (Figure S11).

#### 2.4.2. Evaluation of the Dose-Dependent Effect on L-Gd Encapsulation in hRBCs

Following the preliminary assessment, dose-dependent L-Gd loading experiments were performed. Four different concentrations of the newly synthesized L-Gd complex (2nd batch) were used; subsequently, the biological properties of the cells, as well as the encapsulation efficiency, were assessed under maximal dosing conditions (1–6 mg range, from which derived final L1-, L2-, L3-, L4-Gd RBC samples). For each dose, the L-Gd powder was resuspended in 100 µL of DMSO, and the resulting solutions were used to proceed with the hRBC loading procedure, as described in Section 4. At the end of the loading procedure, all samples were adjusted to a physiological hematocrit, and observations were conducted using TEM, ICP, and NMR measurements. Moreover, the biological parameters of all final RBC samples in the hematocrit range of 40–42% were evaluated using an automated hemocytometer (Table 6).

**Figure 5:**
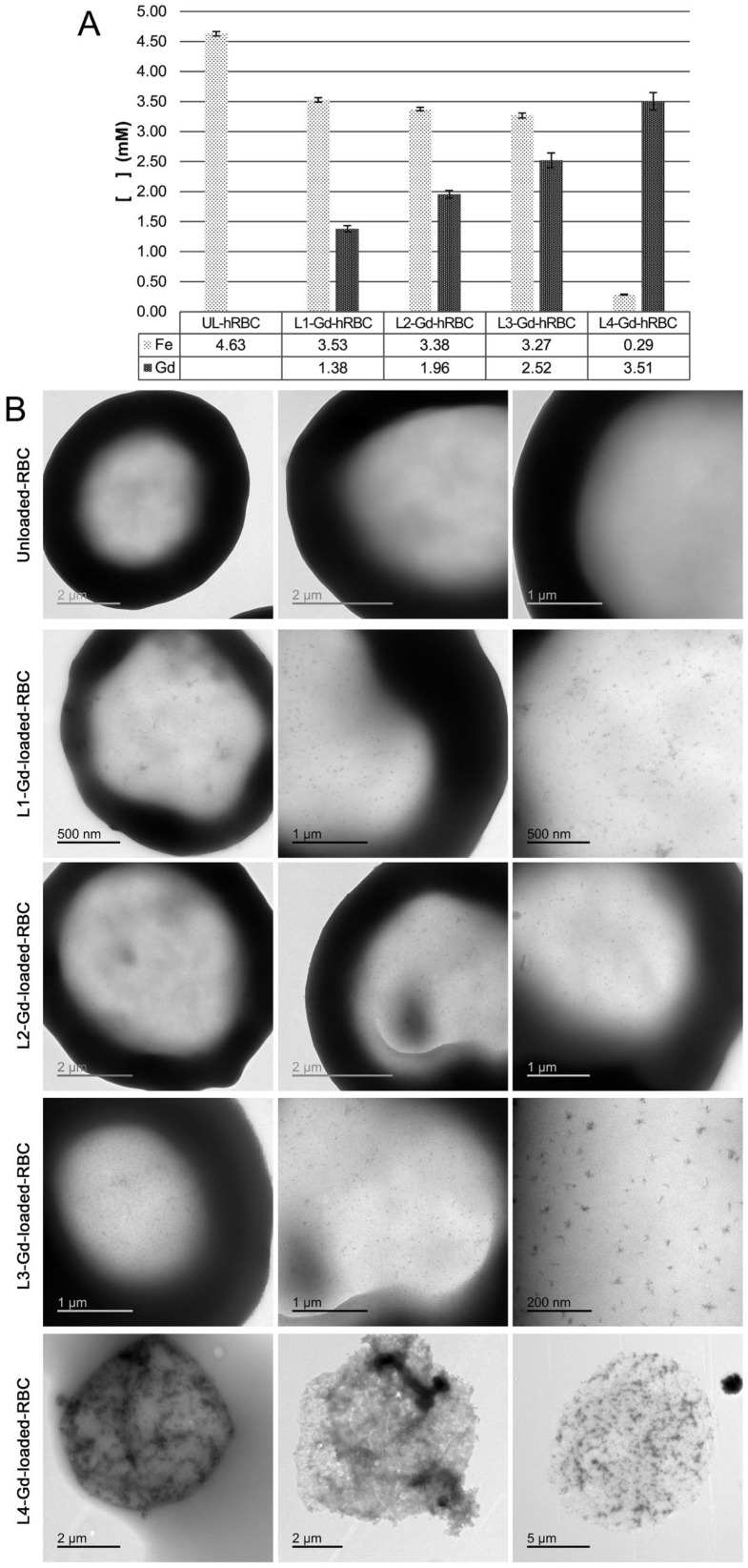
(**A**) ICP-OES analysis of the final L-Gd-loaded RBCs, prepared starting from different initial amounts of the complex. (**B**) Representative TEM images of RBCs loaded with L1–L4 Gd-complexes. The micrographs illustrate the effects of increasing complex concentrations during loading, showing a dose-dependent increase in morphological alterations.

It is evident that in up to certain concentrations of gadolinium complex entrapped into hRBCs, the native cell properties are not significantly altered despite a slight reduction in the Mean Corpuscular Hemoglobin (MCH) value compared to the control group. This variation, which is attributed to the loss of endogenous hemoglobin during the osmotic loading procedure, is consistent with findings previously reported by other authors using similar protocols [39]. Nevertheless, elevated L-Gd loading concentrations result in more significant morphological changes and reduced cell yield (averaging 40–50% compared to unloaded controls), ultimately compromising the physiological profile of the native RBCs. This is manifested as a progressive depletion of hemoglobin, which may eventually become undetectable by automated hematology analysis. Concurrently, ICP quantification of gadolinium revealed a dose-dependent increase in entrapped L-Gd within human RBCs, reaching estimated intracellular concentrations between 1.38 and 3.51 mM (Figure 5). The encapsulation efficiency of L-Gd complex into final human L1–L3 loaded RBCs was 13.5 ± 1.5%, as determined by the ICP-OES quantification of Gd ions. Notably, this efficiency remained constant regardless of the L-Gd dosage up to the L3 level. However, the L4 dose yielded a significantly lower percentage, as the loading process was compromised by impaired physiological morphology and cellular damage. Notably, a significant gadolinium concentration (2.52 mM) was achieved in the hRBC-L3-Gd condition without inducing significant iron loss or alterations in native cell morphology. ICP measurements also showed a concomitant reduction in endogenous iron content, which became more pronounced at the highest L-Gd dose (L4-Gd-RBCs). At this concentration, cell integrity was severely compromised, as evidenced by TEM analysis showing a high prevalence of echinocytes and RBC ghosts. Echinocyte formation may be attributed to metabolic alterations, leading to characteristic membrane deformation (Figure 5).

## 3. Discussion

In conclusion, we have successfully synthesized a novel gadolinium-based complex, L-Gd, comprising a biphenol core and two iminodiacetic side arms capable of coordinating the Gd^3+^ cation through oxygen atoms. The study focused on evaluating its potential for encapsulation within human red blood cells. The advantages of erythrocyte-based delivery systems are well-established, as they effectively maintain active compounds in circulation, enable sustained release, and target specific cells. These carriers are fully biocompatible, offer high loading capacity, and can accommodate small molecules, biologics, and contrast agents [40,41,42,43,44].

Firstly, L-Gd was characterized using analytical techniques such as electron paramagnetic resonance (EPR), which provides a robust spectroscopic protocol for the quantitative evaluation of Gd-based systems and offers valuable insights into the structural features influencing stability and coordination geometry. The EPR study confirmed the validity of the calibration-based procedure for extrapolating complex concentrations and demonstrated clear distinctions between free gadolinium salts and Gd–ligand complexes. The complexes exhibited narrower linewidths, shifted g-values, and enhanced signal intensities, consistent with efficient coordination and structural rigidity. The comparison between g_medium and g_centroid further highlighted the superior symmetry and stability of the complexes relative to the free salt.

Based on the measured EPR parameters, the L-Gd complex synthesized from GdCl_3_ exhibits superior characteristics compared to its sulfate-derived analogue. Furthermore, the EPR data demonstrates that the choice of metal precursor plays a pivotal role in determining the structural integrity and performance of the final complex. The chloride-derived L-Gd complex exhibits higher symmetry, narrower linewidths, stronger and more regular EPR signals, and evidence of more effective coordination, identifying it as the most structurally defined and stable system among those investigated. This study therefore establishes EPR spectroscopy as a robust protocol for the quantitative and qualitative evaluation of Gd-based complexes and supports the chloride-derived L-Gd complex as the most promising candidate for further development and application.

Our dose-dependence studies demonstrated that L-Gd can be efficiently loaded into hRBCs, reaching significant intracellular concentrations ranging from 1.38 to 3.51 mM. While the highest dose (L4-Gd) led to severe morphological alterations and the formation of erythrocyte ghosts, intermediate concentrations (L2-Gd) allowed for substantial gadolinium loading (2.52 mM) without compromising cell integrity or iron content. These findings suggest that L-Gd-loaded RBCs represent a promising and biocompatible platform for diagnostic applications, provided that loading conditions are optimized to preserve the native properties of the carrier cells. Moreover, the proposed method does not suffer from the limitations associated with extracellular Gd agents, which quickly extravasate. Indeed, the long half-life and biocompatibility of Gd-RBCs allow for repeated measurements following a single administration, as these constructs are fully retained within the vascular space. These results are highly relevant in the context of standard clinical doses for Gd-based contrast agents, such as those used in diagnostic imaging. The concentration range of L-Gd that can be encapsulated within hRBCs without inducing significant alterations to their inherent biological properties becomes even more critical when considering that the standard clinical dosage for gadolinium-based contrast agents (e.g., Vasovist^®^) is 2.1 mmol for a 70 kg subject (approx. 0.4 mM). Moreover, a significant advantage of this approach lies in the fact that RBC-encapsulated gadolinium is not subject to renal excretion; L-Gd loaded RBCs remain strictly confined to the intravascular compartment, thereby preventing their extravasation into the interstitial tissues. Theoretically, this allows for the achievement of comparable image quality using a lower total dose than the 2.1 mmol required for free Vasovist^®^. Looking forward, encapsulated L-Gd is expected to act as a ‘blood-pool’ agent. Rather than being excreted by the kidneys within 24 h, it remains in circulation for the entire lifespan of the RBCs (up to 120 days). Moreover, encapsulation serves as a protective shield, preventing gadolinium from accumulating in tissues such as the brain or kidneys, thereby mitigating the risk of nephrogenic systemic fibrosis (NSF). While pioneering studies [2,45] established the feasibility of using RBCs as carriers for Gd-chelates, these investigations did not extend to intracellular imaging or detailed electron microscopy analysis. Future research will be essential to validate this approach using in vivo animal models, specifically to evaluate the circulatory half-life and long-term stability of these novel Gd-RBC constructs. It is hypothesized that once the RBCs are processed, the released Gd-complex undergoes metabolic degradation via the hepatobiliary clearance pathway, as previously observed for similar macromolecular or cell-based systems. Moreover, based on our expertise and previous in vitro and vivo experiments with human SPION-loaded RBCs, we can confirm that encapsulated nanomaterials exhibit a circulation profile identical to that of native RBCs, with primary clearance occurring in the liver and spleen of murine models [46,47].

## 4. Materials and Methods

### 4.1. Synthesis

All chemicals were purchased in the highest quality commercially available. The solvents were RP grade, unless otherwise indicated, and used without further purification. Elemental analysis was performed with a FlashEA^TM^ 1112 EA CHN analyzer, Eager 300 (dedicated software), Thermo Fisher Scientific (Bremen) GmbH Hanna-Kunath-Str. 11 28199 Bremen, Germany. ^1^H- and ^13^C-NMR spectra were recorded on a Bruker Avance 400 instrument (Bruker’s software TopSpin 2.1), operating at 400.13 and 100.61 MHz, respectively, and equipped with a variable temperature controller. The temperature of the NMR probe was calibrated using 1,2-ethanediol as calibration sample. For the spectra recorded in DMSO and D_2_O, ^1^H and ^13^C peak positions are reported with respect to the residual solvent peak. Chemical shifts (δ scale) are reported in parts per million (ppm) and coupling constants (J) are given in Hertz (Hz). IR spectra were recorded on a Shimadzu IRAffinity-1S spectrometer (SHIMADZU Europa GmbH, Duisburg, Germany) equipped with an attenuated total reflectance (ATR) sampling accessory with a diamond crystal (LabSolutions Manager, Release 1.86 SP2).

#### 4.1.1. Synthesis of 3,3′-bis[N,N-bis(Cyanomethyl)aminomethyl]-2,2′-biphenol (3)

Into a three-necked round-bottom flask equipped with a magnetic stirrer and reflux condenser was added iminodiacetonitrile (5.7 g, 43.0 mmol) and paraformaldehyde (2.6 g, 86.0 mmol) in 300 mL of 1,4-dioxane. The reaction was stirred at 100 °C for 30 min after that. Biphenol (1) (4.0 g, 21.5 mmol), dissolved in 100 mL of 1,4-dioxane, was added dropwise, and the reaction was maintained at 130 °C for 40 min. The reaction progress was checked by TLC. The crude reaction was diluted with 100 mL of chloroform and washed with distilled water. Then, the organic layer was separated, dried with anhydrous sodium sulfate and filtered. The solvent was removed under vacuum, and the crude product was crystallized from acetone/chloroform (2:1) to yield (2) (6.8 g, 79%). ^1^H NMR (DMSO) δ = 8.42 (s, 2H), 7.21 (dd, J_1_ = 7.5 Hz, J_2_ = 1.7 Hz, 2H), 7.12 (dd, J_1_ = 7.5 Hz, J_2_ = 1.7 Hz, 2H), 6.93 (t, J= 7.5 Hz, 2H), 3.88 (s, 8H), 3.86 (s, 4H); ^13^C NMR (DMSO) δ = 153.6, 131.9, 130.2, 126.9, 122.9, 120.1, 116.1, 52.8, 41.9 ppm. Anal. Calc. for C_22_H_20_N_6_O_2_ (400.4): C, 66.0%; H, 5.0%; N, 21.0%. Found: C, 66.2°%; H, 5.3%; N, 20.7%. FTIR (ATR): ν (cm^−1^) 3136 (OH stretch), 2259–2242 (CN stretch).

#### 4.1.2. Synthesis of 3,3′-bis[N,N-bis(Carboxymethyl)aminomethyl]-2,2′-biphenol (H_6_L)

In a round bottom flask equipped with a magnetic stirrer and reflux condenser was added 3,3′-bis[N,N-bis(cyanomethyl)aminomethyl]-2,2′-biphenol (2) (4.0 g, 10.0 mmol) and 60 mL of 5 M NaOH. Then, the mixture was refluxed until ammonia production ceased. Afterward, the mixture was cooled down and the white precipitate that formed was filtered. The solid collected was washed with EtOH and dried to afford L as sodium salt Na_5_HL (5.1 g, 90%). ^1^H NMR (D_2_O) δ = 7.27 (d, J = 7.6 Hz, 2H), 7.18 (d, J = 7.2 Hz, 2H), 6.87 (t, J = 7.2°Hz, 2H), 3.96 (s, 4H), 3.32 (s, 8H); ^13^C NMR (CDCl_3_) δ = 176.3, 156.5, 131.4, 130.5, 127.8, 122.7, 118.6, 56.8, 55.5 ppm. Anal. Calc. for C_22_H_19_N_2_Na_5_O_10_ (586.4): C, 45.1%; H, 3.3%; N, 4.8%. Found: C,°45.3%; H, 3.4%; N, 4.7%. FT-IR (ATR): ν (cm^−1^) 3285 (OH stretch), 1576, 1404 (COO stretch).

#### 4.1.3. Synthesis of GdLH_3_

A sample of GdCl_3_ (4.7 mg, 0.018 mmol) in water (0.2 mL) was added to an aqueous solution (0.3 mL) containing Na_4_H_2_L (10.2 mg, 0.018 mmol). The pH of the resulting solution was adjusted to 4 with 0.1 M HCl at room temperature. After a few minutes, a colorless microcrystalline solid precipitated and collected to yield [GdLH_3_]•H_2_O (9.1 mg, 78%). Anal. Calc. for C_22_H_23_GdN_2_O_11_ (648.7): C, 40.7%; H, 3.6%; N, 4.3%. Found: C, 40.8%; H, 3.7%; N, 4.2%. FT-IR (ATR): ν (cm^−1^) 3244 (OH stretch), 1593, 1390 (COO stretch).

### 4.2. Energy Dispersive X-Ray Fluorescence Spectrometry (ED-XRF)

X-ray fluorescence spectrometry analyses were conducted using an Oxford X-Met 8000 Energy Dispersive X-Ray Fluorescence (ED-XRF) spectrometer (Oxoford, UK), which provides information on the elemental composition of the area under investigation. The instrument is equipped with an SSD X-Flash detector, an X-ray tube with a rhodium target anode, and has a 6 mm diameter spot. It can operate at both 8 kV, 50 µA (for low atomic weight elements) and/or 40 kV, 8 µA (for high atomic weight elements): in the first case, the operating condition is particularly sensitive to light elements (starting with Al), while in the second case, information is obtained regarding heavier elements. The measurement time was 120 s, and data were processed using Bruker Artax 7 software. The presence of gadolinium was assessed by ED-XRF analysis through comparison with the reference spectrum of 99% pure gadolinium.

### 4.3. Characterization of L-Gd by Electron Paramagnetic Resonance (EPR) Spectroscopy

Electron paramagnetic resonance (EPR) spectroscopy was employed to investigate the local magnetic environment of Gd^3+^ in the novel L-Gd complex in both DMSO solution and solid state. Gadolinium(III), with a half-filled 4f^7^ electronic configuration (^8^S_7_/_2_, S = 7/2, L = 0), is particularly suitable for EPR investigations due to its high spin multiplicity, giving rise to observable EPR transitions in the X-band (~9.2 GHz). However, fast spin-lattice relaxation and the relatively low natural abundance (~15% each) of the EPR-active isotopes ^155^Gd and ^157^Gd (I = 3/2) often lead to spectra that lack resolved fine or hyperfine structure at room temperature [48].

EPR spectra were recorded at 298 K using a Bruker EMX spectrometer (Bruker, Rhein-stetten, Germany) operating at the X-band (exact microwave frequency 9.297 GHz). Spectra were recorded using WINEPR software (Bruker BioSpin GmbH, Rheinstetten, Germany) as first derivatives using 100 kHz magnetic field modulation, 2 G modulation amplitude, and 10 accumulations to enhance the signal-to-noise ratio. Two types of samples were analyzed: (i) lyophilized solids (GdCl_3_ and L-Gd complex), and (ii) DMSO solutions, including GdCl_3_ at five concentrations (0.2–1.0 mM) and L-Gd complex (0.0014 g dissolved in 300 μL of DMSO, nominal ~4.67 mM).

Data processing was carried out using OriginPro (OriginLab Corporation, North-ampton, MA, USA). Spectral analysis was performed using Gaussian simulations and fitting procedures to extract quantitative EPR parameters. Two complementary approaches were used to define the signal position: (i) g_medium, calculated from B_medium = (B_min + B_max)/2, and (ii) g_centroid, calculated from the spectral centroid B_centroid = ∫ B·I(B) dB/∫ I(B) dB, where B is the magnetic field and I(B) is the absorption intensity obtained after double integration. Peak-to-peak linewidth (ΔB) was determined as ΔB = B_max − B_min. Integrated intensity, proportional to the number of paramagnetic spins, was obtained from the double integral of the derivative spectrum.

A calibration curve was constructed using GdCl_3_ solutions of known concentration and used to estimate the relative Gd^3+^ concentration in the L-Gd complex. The small discrepancy between the EPR-estimated molecular weight and ICP-derived value likely reflects calibration uncertainties and matrix effects in DMSO.

### 4.4. Biocompatibility Testing and L-Gd Loading in Human Red Blood Cells

#### 4.4.1. In Vitro Biocompatibility of L-Gd Complex in HEK293 Cells

HEK 293 cells were obtained from ATCC (LGC Standards S.r.L., Milan, Italy) and cultured in DMEM supplemented with 10% heat-inactivated fetal bovine serum (FBS), 100 U/mL penicillin, and 100 µg/mL streptomycin. Cells were maintained at 37 °C in a humidified atmosphere containing 5% CO_2_. The culture medium was replaced every 2–3 days, and cells were passaged at 70–80% confluence using 0.25% trypsin–EDTA in calcium- and magnesium-free PBS, in 25 cm^2^ culture flasks. Cell viability was determined using a Vi-CELL BLU automated cell counter (v1.4.3.1; Beckman Coulter, Brea, CA, USA) 24 h after L-Gd treatment. The L-Gd complex (1.4 mg) was dissolved in 50 µL of DMSO and added to HEK 293 cells cultured under standard conditions in a total medium volume of 8 mL. In this condition, the final percentage value of DMSO is safe for cell culture and widely accepted in experimental practice.

#### 4.4.2. Loading of L-Gd Complex in Human Red Blood Cells (hRBCs)

The encapsulation of the L-Gd complex in human RBCs was investigated by testing different doses of the compound to determine entrapment efficiency. The loading procedure involved dissolving the L-Gd powder in DMSO, using a range of 1.4 mg to 6 mg to assess the process in a dose-dependent manner. For investigations using human blood, informed consent was obtained from the participants involved, on the basis of an official document of accordance with the Transfusion Center of “S. Maria della Misericordia” Hospital in Urbino (PU), Italy. Human blood was collected from healthy volunteers into heparinized tubes. RBCs were isolated by centrifugation at 1400× *g* at 4 °C for 10 min from freshly drawn blood. The serum and buffy coat were removed, and the packed cells were washed three times with HEPES buffer (10 mM HEPES, 140 mM NaCl, 5 mM glucose, pH 7.4) and then resuspended in the same buffer with 70% hematocrit. These cells were dialyzed in the presence of L-Gd complex solution for 75 min using a tube with a 12–14 kDa cut-off in 50 vol of a dialysis buffer (10 mM NaHCO_3_, 10 mM NaH_2_PO_4_, 20 mM glucose, and 4 mM MgCl_2_ at pH 7.4), containing 2 mM ATP and 3 mM reduced glutathione. The osmolarity of the dialysis buffer was 70 mOsm. Resealing of RBCs was achieved by adding 0.1 vol of PIGPA (5 mM adenine, 100 mM inosine, 2 mM ATP, 100 mM glucose, 100 mM sodium pyruvate, 4 mM MgCl_2_, 194 mM NaCl, 1.606 M KCl, and 35 mM NaH_2_PO_4_ at pH 7.4) per vol of dialyzed RBCs and by incubating at 37 °C for 45 min. The resealed cells were recovered by centrifugation at 400× *g* and washed four times with HEPES buffer to remove the untrapped L-Gd compound. Finally, hematological indices, including mean corpuscular volume (MCV), mean corpuscular hemoglobin (MCH), and mean corpuscular hemoglobin concentration (MCHC), were examined by a hemocytometer (ABX MICROS ES 60 OT, HORIBA ABX SAS, Montpellier Cedex4, France) to evaluate the cellular integrity of the L-Gd-loaded RBCs compared to the control samples (Unloaded-RBCs) at the same hematocrit percentage. The morphology and intracellular features of the final L-Gd loaded RBCs were studied using a transmission electron microscope (TEM).

### 4.5. Transmission Electron Microscopy (TEM)

A transmission electron microscope (TEM) JEM-1011 (JEOL Ltd., Akishima, Tokyo, Japan) operating at an accelerating voltage of 100 kV was used to analyze unloaded- and -L-Gd loaded RBCs after their fixation with 2.5% glutaraldehyde and by dropping a few microliters of each sample onto a formvar-coated copper grid as previously reported [41]. In detail, 5 μL of glutaraldehyde-fixed RBCs (1–4% Ht) was dropped onto a formvar-coated copper grid (placed on a parafilm) and left to react for 5 min; after that, the grid was washed twice upside down on a 50 μL drop of water for 1 min; finally, the excess liquid was removed for capillarity with a Whatman filter paper (type 1).

### 4.6. ESEM-EDS Analysis

Environmental scanning electron microscopy (ESEM) was performed using an FEI Quanta 200 FEG (FEI, Hillsboro, OR, USA) equipped with an energy-dispersive X-ray spectrometer (EDS) (EDAX Inc., Mahwah, NJ, USA). As an evolution of conventional SEM, ESEM enables the imaging of non-conductive samples without the need for gold or carbon coating, regardless of vacuum levels. This is particularly advantageous for biological specimens, as it allows for morphological analysis without prior treatment. When integrated with EDS, ESEM facilitates semi-quantitative elemental detection of ultrastructural components through point or area analysis. This preserves the high spatial resolution of morphological imaging while avoiding the artifacts or interferences typical of sample preparation in conventional SEM. For this study, aliquots of L-Gd powder or 10–15 µL of human RBC samples fixed in 2.5% glutaraldehyde were deposed on aluminum stubs covered with carbon conductive adhesive discs (TAAB Ltd., Berks, UK). Analyses were performed at least 48 h after air drying (only for hRBCs) using an electron beam focused on an electron gun at a pressure of 5.0 × 10^−6^ mbar. The ESEM operated in low-vacuum mode with a chamber pressure of 0.80 mbar, an accelerating voltage from 25 to 30 kV, a working distance of 10 mm, and a spot size of 3.9. Data were collected at a 0° tilt angle with a 30–33% dead time and an acquisition time from 100 to 500 s. Images were obtained using a backscattered electron detector or secondary electron detector to highlight the presence of Gd in the sample [49].

### 4.7. ICP Analysis

Metal content (Fe or Gd) was obtained by ICP-OES. The metal concentrations were measured by elemental analysis using an inductively coupled plasma atomic emission spectrometer (720 ICP-OES, Agilent Technologies/Varian, Palo Alto, CA, USA). Before analysis, 30 µL of the samples was digested with 800 µL of a concentrated solution (HCl/HNO_3_/H_2_H_2_ 3:1:1) for 48 h. After that, the solutions were diluted with UP water up to 6 mL and filtered with 0.44 um nylon filters.

### 4.8. NMR Measurements

The longitudinal (T1) and transverse (T2) relaxation times of the samples at the hematocrit specified in the manuscript were measured at a magnetic field strength of 9.4 Tesla (corresponding to a proton resonance frequency of 400 MHz) at 37 °C using a Bruker AC-400 NMR spectrometer (BRUKER BIOSPIN AG, Faellanden, Switzerland). T1 was determined using a 180-Ƭ-90 inversion recovery sequence with a fixed relaxation delay of at least 5 × T1. The times of inversion (T) were chosen on the basis of an estimated T1 value. T2 was measured using the Carr–Purcell–Meiboom–Gill (CPMG) sequence. The echo-times were chosen on the basis of an estimated T2 value. All data were processed using TopSpin to calculate the relaxation rates.

## Figures and Tables

**Table 2:** EPR spectral parameters of GdCl_3_ reference solutions at various concentrations, the L-Gd complex in DMSO (gray-shaded area), and solid-state samples (s). Reported parameters include the medium (B_medium) and centroid field (B_centroid), corresponding g values, peak-to-peak linewidth (ΔB), and double-integrated signal intensity. All spectra were recorded at 298 K under identical instrumental conditions to allow for quantitative comparison. n.d. = not determined; (s) indicates solid-state measurements.

Samples	B_Medium (G)	g_Medium	B_Centroid (G)	g_Centroid	ΔB (G)	Integrated intensity (a.u.)
GdCl_3_ 0.2 mM	3211.53	2.0683	3133.27	2.1200	385.93	3.41 × 10^6^
GdCl_3_ 0.4 mM	3210.31	2.0691	3093.01	2.1476	383.49	3.41 × 10^6^
GdCl_3_ 0.6 mM	3211.53	2.0683	3141.65	2.1143	385.93	4.04 × 10^6^
GdCl_3_ 0.8 mM	3212.75	2.0675	3153.47	2.1064	388.37	4.23 × 10^6^
GdCl_3_ 1.0 mM	3212.75	2.0675	3154.60	2.1056	388.37	4.30 × 10^6^
L–Gd	3242.06	2.0488	3179.99	2.0888	779.19	1.05 × 10^7^
GdCl_3_ (s)	3239.62	2.0504	3225.49	2.0591	2322.91	—
L–Gd (s)	3210.31	2.0691	3209.94	2.0704	1482.66	—

**Table 3:** Hematological parameters of human RBCs loaded with two different batches (1st and 2nd) of L-Gd complex versus unloaded control cells (UL-RBCs).

Sample	RBCs 10^6^/μL	HGB gr/dL	HCT %	MCV fL	MCH pg	MCHC gr/dL	T1 (ms)	T2 (ms)
UL-RBCs	6.22	12.7	46	77	22.4	29	2079 ± 26.6	58.0 ± 5.9
1st batch L-Gd	6.23	9.0	43	71	15.5	21.8	75.7 ± 4.1	17.54 ± 3.9
2nd batch L-Gd	6.38	8.9	45	70	14.0	20.1	71.1 ± 5.7	8.88 ± 0.87

**Table 4:** Hematological parameters and T1 and T2 relaxation times of L-Gd-loaded and ligand-loaded RBC samples compared to the unloaded (UL-RBCs) control cells.

Samples	RBCs 10^6^/μL	HGB gr/dL	MCV fl	MCH pg	MCHC gr/dL	T1 (ms)	T2 (ms)
UL-RBCs	5.45 ± 0.3	8.26 ± 0.4	70 ± 1.0	13.6 ± 0.4	21.5 ± 0.36	2090 ± 160	100.4 ± 10.6
Ligand-loaded RBCs	6.42 ± 0.16	8.0 ± 0.25	64 ± 1.0	13.3 ± 0.4	21.4 ± 0.26	2020 ± 113	81.8 ± 9.6
L1-Gd-loaded RBCs	5.59 ± 0.17	6.7 ± 0.2	69 ± 1.0	12.8 ± 0.20	19.3 ± 0.20	56.37 ± 4.1	6.54 ± 1.4
L2-Gd-loaded RBCs	4.45 ± 0.10	4.63 ± 0.25	65 ± 1.0	11.0 ± 0.26	15.4 ± 0.43	43.82 ± 7.7	10.2 ± 1.3

**Table 5:** Semi-quantitative ESEM-EDS analysis of atomic and weight percentages for Gd and Fe in human RBCs loaded with L1-Gd and L2-Gd complexes compared with ligand-loaded and unloaded RBCs. Data are expressed as the mean ± SD of five independent acquisitions performed on each sample under identical analytical conditions.

	UL-RBCs		Ligand-RBCs		L1-Gd-RBCs		L2-Gd-RBCs	
	Wt%	At%	Wt%	At%	Wt%	At%	Wt%	At%
Gd	-	-	-	-	0.40 ± 0.22	0.04 ± 0.02	0.62 ± 0.21	0.05 ± 0.02
Fe	0.18 ± 0.06	0.05 ± 0.02	0.16 ± 0.05	0.04 ± 0.01	0.16 ± 0.06	0.04 ± 0.02	0.09 ± 0.04	0.02 ± 0.01

**Table 6:** Evaluation of biological parameters and T1 and T2 relaxation time measurements of loaded hRBCs (L1–L4) compared to the control cells (UL-RBCs). (n.d.: not detectable). * Note: Approximately half of the cells were identified as ghost RBCs, as confirmed by subsequent TEM analysis.

Sample	RBCs (10^6^/µL)	HGB (g/dL)	MCV (fL)	MCH (pg)	MCHC (g/dL)	T1 (ms)	T2 (ms)
UL-RBCs	5.94 ± 0.05	10.7 ± 0.43	69 ± 1.0	16.8 ± 0.35	26.2 ± 0.26	2034 ± 74.6	65.6 ± 4.87
L1-Gd-RBCs	5.39 ± 0.18	8.6 ± 0.2	70 ± 1.5	16.0 ± 0.2	22.6 ± 0.30	84.1 ± 1.91	9.15 ± 0.67
L2-Gd-RBCs	4.97 ± 0.06	8.0 ± 0.25	73 ± 0.5	15.7 ± 0.1	21.5 ± 0.25	63.0 ± 3.1	7.73 ± 0.40
L3-Gd-RBCs	4.93 ± 0.14	7.4 ± 0.36	76 ± 1.0	14.9 ± 0.2	19.7 ± 0.1	46.72 ± 3.3	5.64 ± 0.11
* L4-Gd-RBCs	2.91 ± 0.08	n.d.	81 ± 1.0	n.d.	n.d.	30.53 ± 1.0	6.85 ± 1.8

## Data Availability

Data are contained within the article.

## Supplementary Materials

The following supporting information can be downloaded at: https://www.mdpi.com/article/10.3390/ijms27083492/s1.
