# Supplementary material for: Synthesis and Characterization of a Novel Biphenol-Based Gadolinium Complex for Encapsulation in Human Red Blood Cells
Source: Int J Mol Sci. 2026 Apr 14;27(8):3492. doi: 10.3390/ijms27083492 (PMC13116415; doi:10.3390/ijms27083492)
Supplement: Supplementary file 1 [file ijms-27-03492-s001.zip › ijms-4198047-supplementary.pdf]

[Manuscript ID: ijms-4198047]:

Supplementary Materials for

Synthesis and Characterization of a Novel Biphenol-Based Gadolinium Complex for Encapsulation in Human Red Blood Cells

Antonella Antonelli [1,†,*], Riccardo Di Corato [2,3†], Luca Mancini [1], Michela Cangiotti [4], Laura Valentini [1], Luca Giorgi [4], Gianluca Ambrosi [4], Pietro Gobbi [1], Erika Palazzetti [4], Luigia Rossi [1], Mauro Magnani [1]

[1] Dipartimento di Scienze Biomolecolari (DISB), Università degli Studi di Urbino Carlo Bo, Campus Scientifico "E. Mattei", Via Cà Le Suore 2-4, Urbino 61029 (PU), Italy; antonella.antonelli@uniurb.it (A.A.); luca.mancini@uniurb.it (L.M.); laura.valentini@uniurb.it (L.V.); pietro.gobbi@uniurb.it (P.G.); luigia.rossi@uniurb.it (L.R.); mauro.magnani@uniurb.it (M.M)

[2] Institute for Microelectronics and Microsystems (IMM), CNR, Via Monteroni, Lecce 73100; riccardo.dicorato@cnr.it (R.D.C.)

[3] Center for Biomolecular Nanotechnologies, Istituto Italiano di Tecnologia, Arnesano, Lecce 73100, Italy.

[4] Dipartimento di Scienze Pure e Applicate (DiSPeA) Urbino 61029 (PU), Italy; michela.cangiotti@uniurb.it (M.C.); luca.giorgi@uniurb.it (L.G.); gianluca.ambrosi@uniurb.it (G.A); erika.palazzetti@uniurb.it (E.P.)

*Author to whom correspondence should be addressed.
[†]These authors contributed equally to this work.

Synthesis of the gadolinium complex. While ^{1}H and ^{13}C NMR spectra details are provided in the manuscript, the corresponding spectra for all compounds synthesized and shown in Figure 1 (related to the synthesis of the gadolinium complex GdH_3L, designated as L-Gd), are presented here in Figure S1.

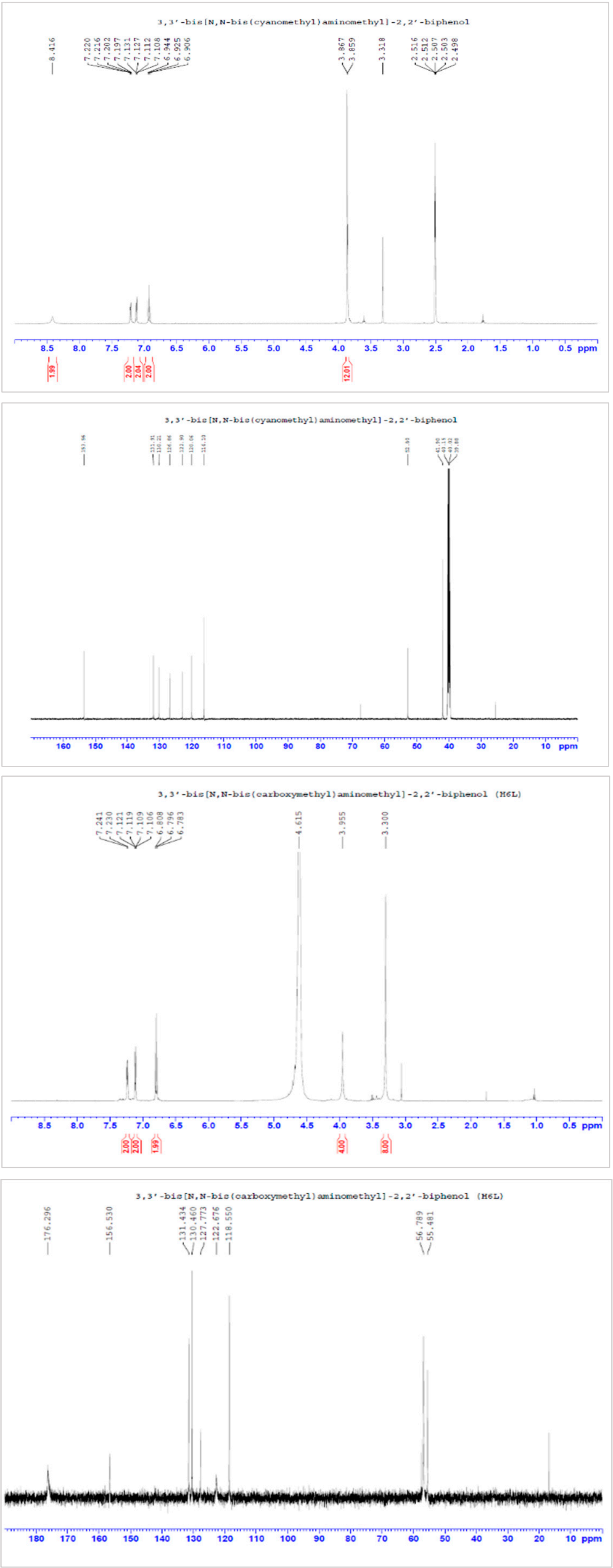

Figure S1. ^{1}H and ^{13}C NMR spectra of all the compounds synthesized and presented in Figure 1

IR spectroscopic study. FT-IR analyses of the GdH_3L complex (hereafter referred to as L-Gd) was sterilized at 120°C for 24 hours to assess its functionality in a cell-based drug delivery model, specifically using human red blood cells (hRBCs) under sterile conditions. FT-IR spectroscopy was performed on two samples: one before and one after sterilization. The overlaid spectra were identical, indicating that the complex remained intact and confirming its thermal stability, Figure S2.

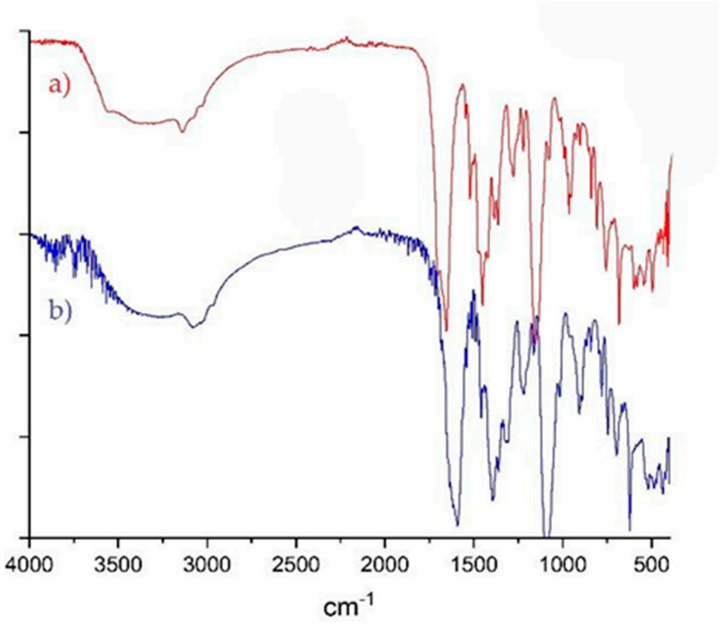

Figure S2. IR spectra of the GdH_3L complex before (a) and after (b) sterilization at 120 °C. The overlapping profiles indicate structural integrity and confirm the thermal stability of the complex.

XRF measurements. Characteristic gadolinium peaks were identified in the ED-XRF spectra of $GdCl_3$ and $Gd_2(SO_4)_3$ powders, as well as in the L-Gd complex synthesized from the chloride salt. These assignments were further validated by comparison with a high-purity (99%) gadolinium reference spectrum (Figure S3).

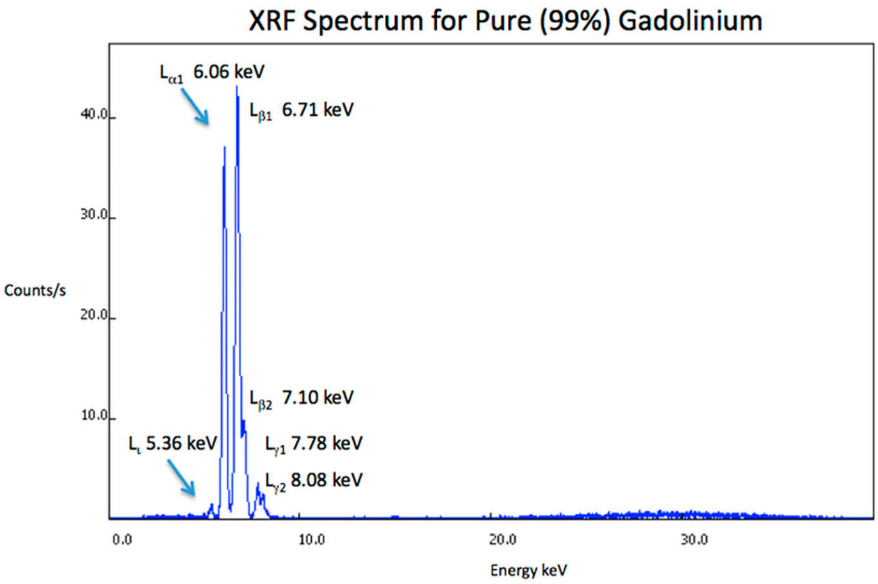

Figure S3. ED-XRF Spectrum of pure Gadolinium, taken with an Innov-X a-2000 X-Ray Fluorescence spectrometer with a Si-PiN detector (Hardware settings: Source: Ta; Voltage: 40 kV; Current: 24 μA; Filter: 250 μM Cu, Analytical. Available at: https://www.xrfresearch.com/xrf-spectrum-gadolinium/

EPR measurements. This section contains additional EPR data supporting the main text, including calibration curves and comparison of L-Gd complexes prepared from different Gd salts (Figure S4).

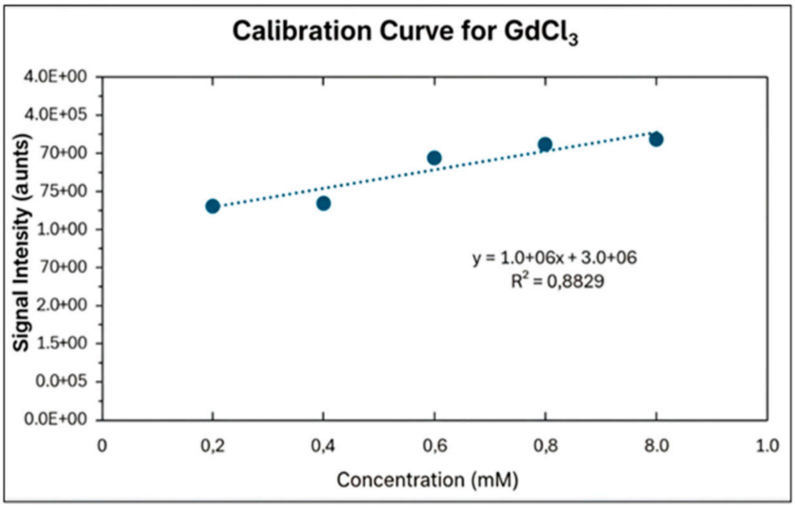

Figure S4. Calibration curve of X-band EPR signal intensity versus concentration for $GdCl_3$ solutions in DMSO (0.2-1.0 mM). Integrated intensities (a.u.) were obtained under identical instrumental conditions at 298 K. The curve was used to estimate the concentration of EPR-active Gd^{3+} in L–Gd solutions.

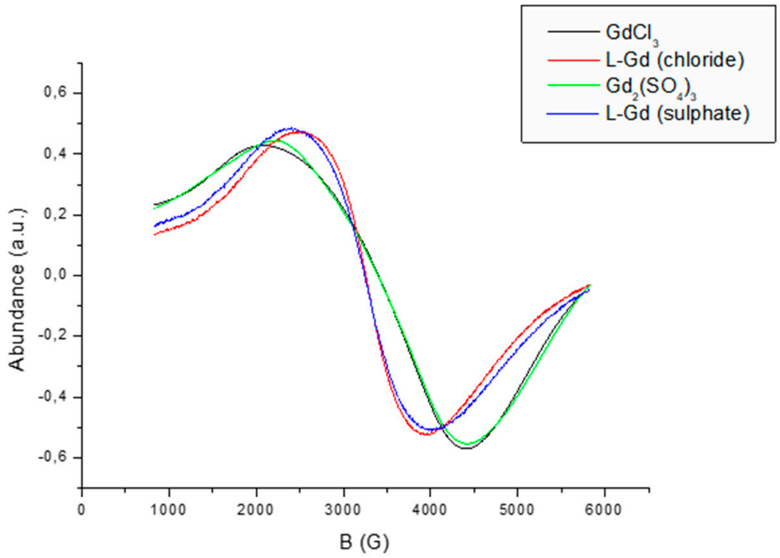

Figure S5. Normalized X-band EPR spectra of $GdCl_3$, $Gd_2(SO_4)_3$, and L–Gd complexes prepared from both Gd salts and recorded in the solid state at 298 K. Spectra were normalized to the same peak intensity to allow direct comparison of line shape, linewidth, and anisotropy. The x-axis represents the magnetic field (G), and the y-axis the first-derivative EPR signal intensity (a.u.). This comparison highlights differences in coordination environment and spectral symmetry depending on the counterion.

ESEM-EDS analysis of GdCl3 and the L-Gd complex. Representative ESEM-EDS images and corresponding spectra of reproducible L-Gd powders (synthesized from $GdCl_3$) are shown in Figure S6. Additionally, the spectra of the $GdCl_3$ precursor used for the synthesis are reported in Figure S7.

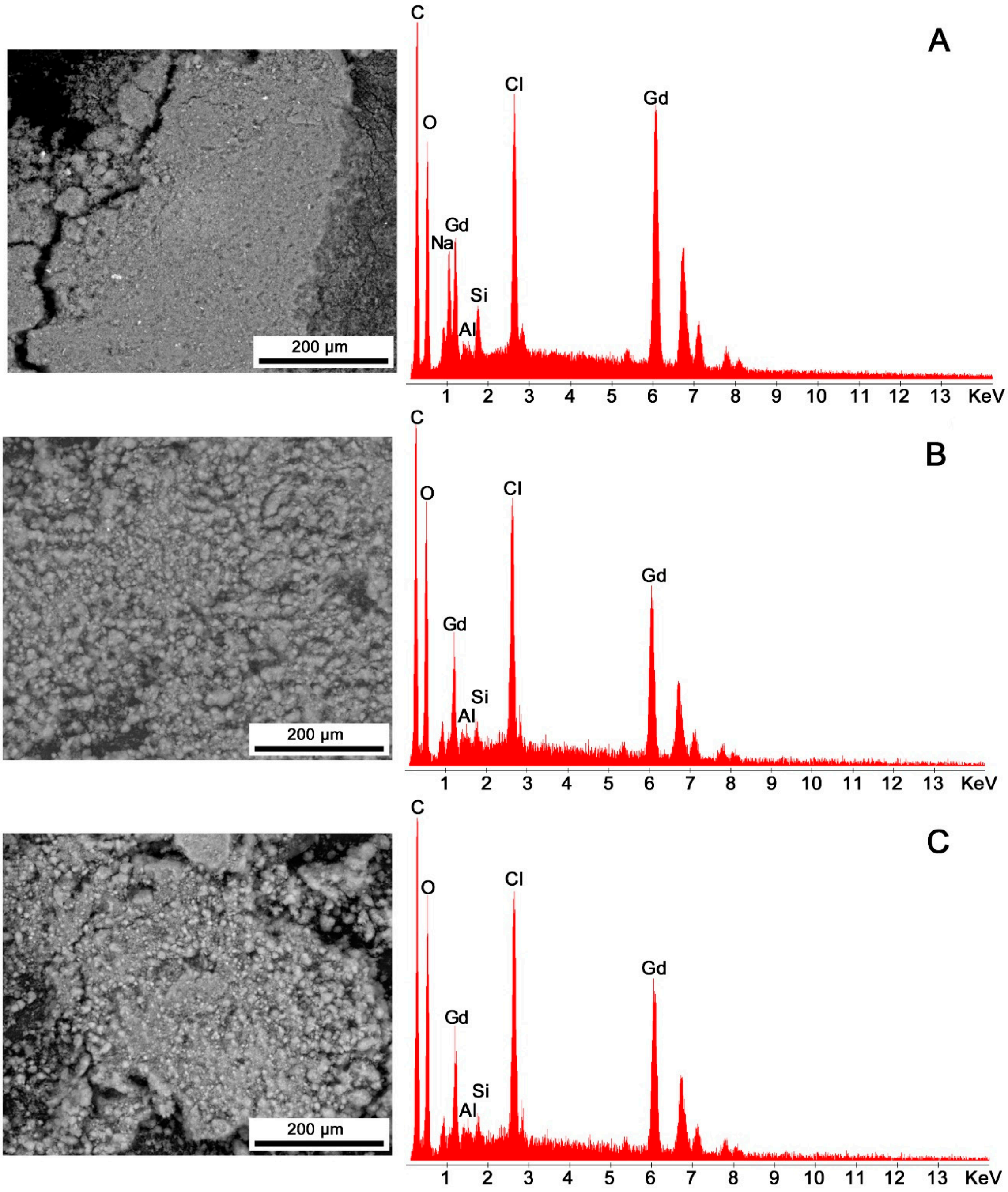

D

Sample	1st L-Gd		2nd L-Gd		st. 2nd L-Gd	
Elem	Wt%	At%	*Wt%*	*At%*	*Wt%*	*At%*
C	48.04	69.28	47.32	70.71	51.55	71.22
N	3.72	4.57	-	-	4.16	5.00
O	20.10	21.72	18.86	20.94	21.60	22.37
Cl	4.90	2.40	3.32	1.72	4.45	2.10
Gd	22.98	2.54	25.31	3.03	19.56	2.08

Figure S6. ESEM-EDS images and corresponding spectra of L-Gd compound (2nd batch) (**A**) synthesized on the basis of synthesis protocol used for the first preparation (1st batch) of L-Gd complex (**B**). Image and spectrum reported in panel (**C**) correspond to sterilized L-Gd complex. The sterilization process did not affect the chemical ratios of sample C, confirming the chemical stability of the preparation. The images were acquired at the same magnification. The EDS analyses were performed under the same analytical conditions. (**D**) The values of major elements present in L-Gd powder samples represent the means of ten independent acquisitions performed on each sample under identical analytical conditions (e.g., incident beam energy, acquisition time, analysis area, dead time, and working distance). Given the semi-quantitative nature of the analysis, results should be interpreted based on elemental ratios rather than absolute values.

ESEM-EDS analysis of $GdCl_3$. Representative environmental scanning electron microscopy (ESEM) images and energy-dispersive X-ray spectroscopy (EDS) spectra of the $GdCl_3$ powder used for the synthesis of the L-Gd complex (Figure S7).

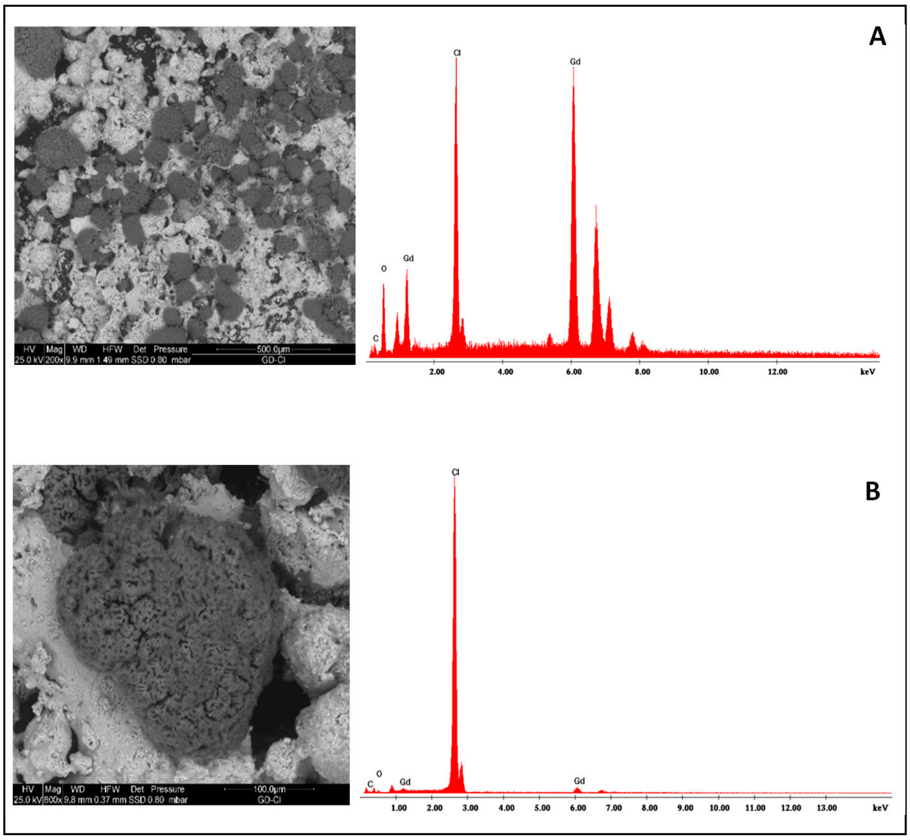

Figure S7. ESEM-EDS analyses of $GdCl_3$ powder alongside the corresponding spectrum. A) Image at low magnification and spectrum obtained by analyzing a small area of the sample, B) Image at higher magnification and spectrum acquired from the dark area.

Preliminary biocompatibility assessment of L-Gd complexes with human RBCs (hRBCs)

1. ***$GdCl_3$ addition to hRBCs***

The impact of gadolinium on human red blood cells (hRBCs) integrity by incubating the cells with either free $GdCl_3$ or the L-Gd complex was evaluated. Human blood was collected from healthy volunteers into heparinized tubes. hRBCs were isolated from freshly drawn blood by centrifugation at 1400 × g for 10 min at 4 °C. After removal of the plasma and buffy coat, the packed cells were washed three times with HEPES buffer (10 mM HEPES, 140 mM NaCl, 5 mM glucose, pH 7.4) and resuspended in the same buffer to a final hematocrit of 70% (mimicking %HCT condition applied during the loading protocol). An aliquot of $GdCl_3$ (MW 263.61) dissolved in 100 µL Hepes buffer was added to 1 mL of the RBC suspension. The final osmolality of the suspension was 280 mOsm similarly to standard physiological. Cellular biological parameters were evaluated using a hemocytometer at t= 0 and t=3h after incubation. As shown in **Table 1S**, the native RBC parameters did not exhibit significant changes after 3 h of incubation with $GdCl_3$. Table 1S also reports the NMR longitudinal T1 and transverse T2 relaxation times measured at the end of the 3-hour incubation period, before and after washing of cell suspension to evaluate the overall paramagnetic effect in the samples.

Table S1. Evaluation of the biological properties of human red blood cells (RBCs) treated with $GdCl_3$ and NMR measurements performed at the end of the incubation period (3 h). Values of untreated control RBCs were also reported. **T1 and T2 values were also measured after washing the RBCs with HEPES buffer and resuspending them to a final hematocrit of approximately 70%.*

Samples	RBCs 10^6/µl	HGB gr/dL	HCT %	MCV fL	MCH pg	MCHC gr/dL	T1 (ms)	T2 (ms)
Control RBCs	7.45±0.08	23.1±0.3	69±1.0	92±1.0	30.8±0.6	33.2±0.4	1781±17.6	48.9±1.9
RBCs+$GdCl_3$ t=0h	6.90±0.15	19.7±0.40	69.5±0.9	90.6±1.5	29.0±0.8	32.5±0.8	-	-
RBCs+$GdCl_3$ t=3h	6.25±0,29	19.4±0.25	66.5±0.6	92±1.0	30.6±0.7	33.1±0.8	429.72±43.8 *581.9±32.4	37.98±2.0 *48.49±7.0

Transmission electron microscopy (TEM) analyses were performed on the same samples and representative images of human RBCs incubated with $GdCl_3$, either washed (A) or unwashed (B) with HEPES buffer, are shown in Figure S8.

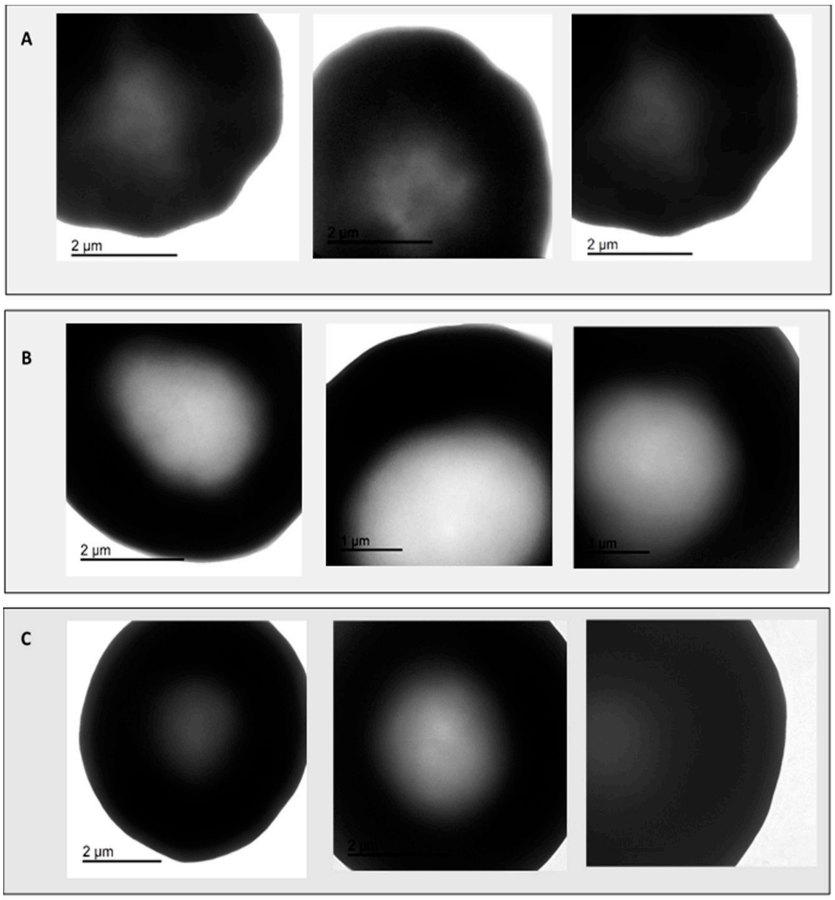

Figure S8. TEM analysis of human RBCs incubated with $GdCl_3$. (A) RBCs incubated with $GdCl_3$ and immediately washed twice with HEPES buffer. (B) RBCs incubated with $GdCl_3$ without washing. (C) Untreated human RBCs (control cells).

2. *L-Gd complex addition to hRBCs*

A total of 1.4 mg of L-Gd (dissolved in 50 µL of pure DMSO) was added to an RBC suspension (in HEPES buffer, for a total volume of 6.5 mL) at a final hematocrit of 20% to ensure improved and homogeneous dispersion within the cell suspension. The suspension was maintained at room temperature under gentle agitation for 3 hours. Immediately afterward, cell integrity was evaluated using standard RBC parameters measured with a hemocytometer.

The experimental results are summarized in **Table S2**, together with the relaxation times measured via 400 MHz NMR spectroscopy; these values served to confirm the paramagnetic effect of gadolinium, as evidenced by the significant reduction in relaxation rates compared to control samples. Additionally, the impact of L-Gd on whole blood (w.b.) was investigated at a physiological hematocrit (44%), revealing no significant alterations in the intrinsic native biological properties of the erythrocytes. These results were further supported by transmission electron microscopy (TEM) analysis (Figure S9), which demonstrated that L-Gd-treated RBCs maintained a characteristic biconcave morphology (Figure S9A) with no detectable ultrastructural aberrations, either intracellularly or extracellularly, relative to untreated controls (Figure S9B).

Table S2. Hematological parameters and relaxation times of human RBCs in the presence of the L-Gd complex.

**Samples**	**RBCs** $10^6/\mu l$	**HGB** gr/dL	**HCT** %	**MCV** fL	**MCH** pg	**MCHC** gr/dL	**T** (ms)	**T2** (ms)
w.b.	5.94±0.11	15.5±0.6	45.3±2.3	88±1.0	29.3±0.3	32.4±0.8	1968±50.7	55.75±6.7
w.b. + L-Gd t=3h	6.08±0.21	16.6±0.5	45.8±1.2	90±1.0	29.8±0.4	32.5±1.0	894±18.3	45.4±9.0
Untreated-RBCs	2.74±0.08	5.7±0.65	19.6±0.6	90±1.0	23.2±1.3	32.7±0.6	3481±0.6	267.1±58
L-Gd + RBCs t=0h	2.36±0.15	5.3±0.45	21±0.9	92±2.0	21.5±0.6	25.03±2.5	1389±20.5	247.3±41.4
L-Gd + RBCs t=3h	2.04±0.05	5.16±0.2	19.4±0.2	90±1.0	23.6±0.8	27.3±0.4	1419±20.5	194.7±4.6

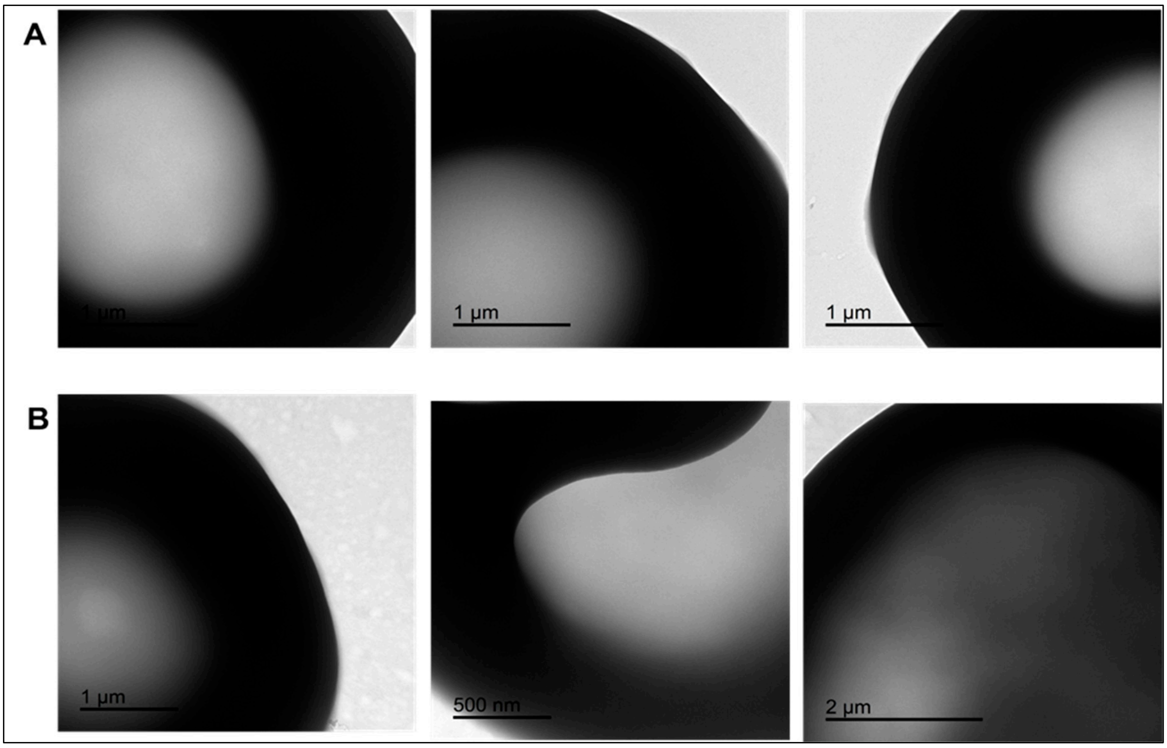

Figure S9. TEM images of human RBCs after L-Gd incubation (**A**) and untreated control RBCs (**B**).

ICP and TEM analyses of hRBCs loaded with ligand alone, L1-Gd and L2-Gd. Quantitative ICP measurements showed no significant alterations in intracellular iron levels following ligand loading (3.76 ± 0.09 mM vs. 3.32 ± 0.08 mM in unloaded hRBCs). Additionally, Gd levels remained undetectable in these samples, consistent with the experimental design.

In contrast, ICP analysis confirmed a successful Gd uptake in L-Gd-loaded RBCs; specifically, the Gd concentration in L2-Gd samples was approximately twofold higher than in L1-Gd samples (3.43 ± 0.11mM vs. 1.73 ± 0.08 mM, respectively, respectively; Figure S10).

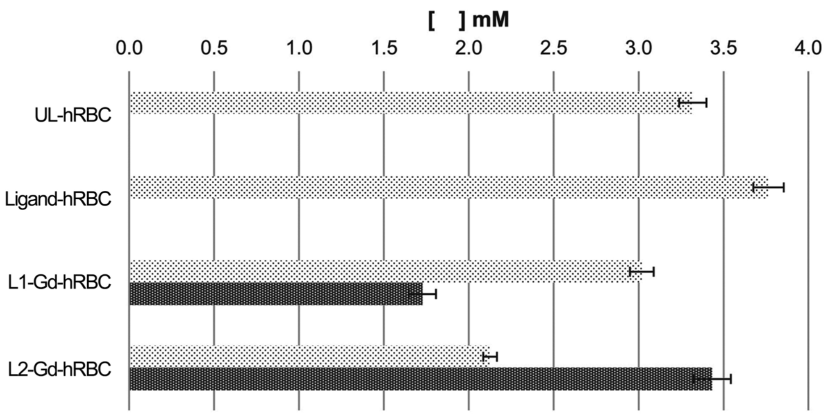

	UL-hRBC	Ligand-hRBC	L1-Gd-hRBC	L2-Gd-hRBC
Fe	3.32 ± 0.08	3.76 ± 0.09	3.02 ± 0.07	2.13 ± 0.04
Gd	---	---	1.73 ± 0.08	3.43 ± 0.11

Figure S10. ICP determination of metal content in L1-Gd- and L2-Gd-loaded RBCs compared to Ligand-loaded and Unloaded-RBCs.

TEM analysis confirmed that the encapsulation of the ligand alone did not induce significant morphological or intracellular alterations, with the ultrastructural integrity of the hRBCs remaining comparable to that of unloaded control cells, Figure S11.

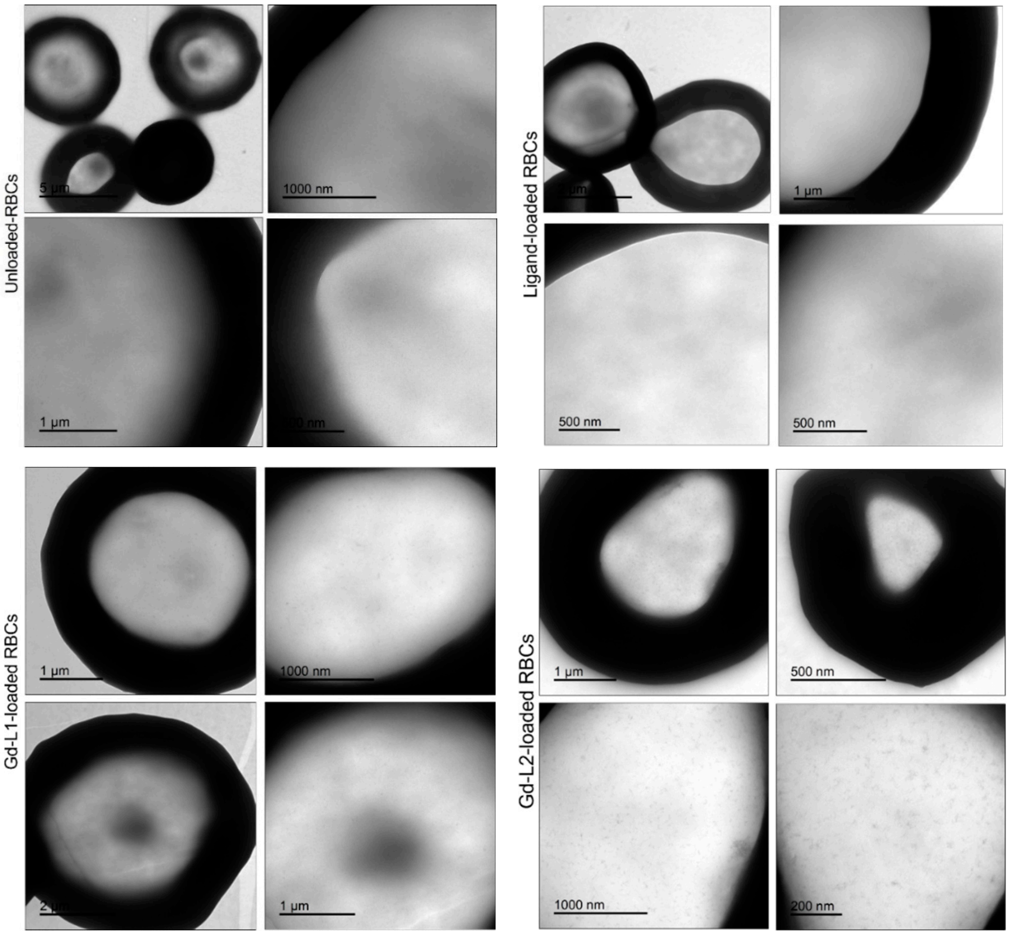

Figure S11. To assess potential structural changes, TEM analysis was performed on ligand-loaded, L1-Gd-loaded, and L2-Gd-loaded RBCs, using unloaded cells as a reference control.
